# Bridging the gap in OA therapeutics: Bioengineered strategies to target osteoclast–chondrocyte crosstalk

**DOI:** 10.1002/btm2.70107

**Published:** 2026-01-11

**Authors:** Enbo Zhang, Chi Ma, Xiaohe Fan, Bowen Gu, Bo Li

**Affiliations:** ^1^ Department of Orthopedics III Qiqihar Traditional Chinese Medicine Hospital Qiqihar China; ^2^ Department of Orthopedics I QTH Coal General Hospital Qitaihe China; ^3^ Department of Psychiatry and Psychology Hongqi Hospital Affiliated to Mudanjiang Medical University Mudanjiang China; ^4^ Department of Rehabilitation Medicine Qiqihar Traditional Chinese Medicine Hospital Qiqihar China; ^5^ Department of Rheumatology and Immunology Hongqi Hospital Affiliated to Mudanjiang Medical University Mudanjiang China

**Keywords:** nanomedicine, osteoarthritis, osteoclast‐chondrocyte communication, small‐molecule inhibitors, targeted drug delivery

## Abstract

Osteoarthritis (OA) is a degenerative joint disease characterized by cartilage degradation, subchondral bone remodeling, and joint microenvironment imbalance. Emerging evidence identifies pathological osteoclast–chondrocyte crosstalk as a key OA driver, mediated through RANKL/RANK/OPG, NF‐κB, HIF‐2α, and VEGF signaling pathways that create a destructive bone–cartilage feedback loop. This review examines: (1) molecular mechanisms underlying this cellular communication, (2) therapeutic small‐molecule inhibitors targeting CatK, MMP‐13, NFATc1, and Runx2, and (3) innovative nanomedicine approaches including tissue‐specific nanoparticles, smart delivery systems, and combination therapies. We evaluate these strategies' preclinical validation in animal and organoid models while addressing translational challenges in biosafety, tissue targeting, and personalized delivery. By integrating intercellular signaling knowledge with advanced therapeutic technologies, we provide a framework for developing disease‐modifying OA treatments that bridges basic research with clinical precision medicine applications.

## Graphical abstract

Translational roadmap of OA nanotherapeutic platforms toward clinical application.

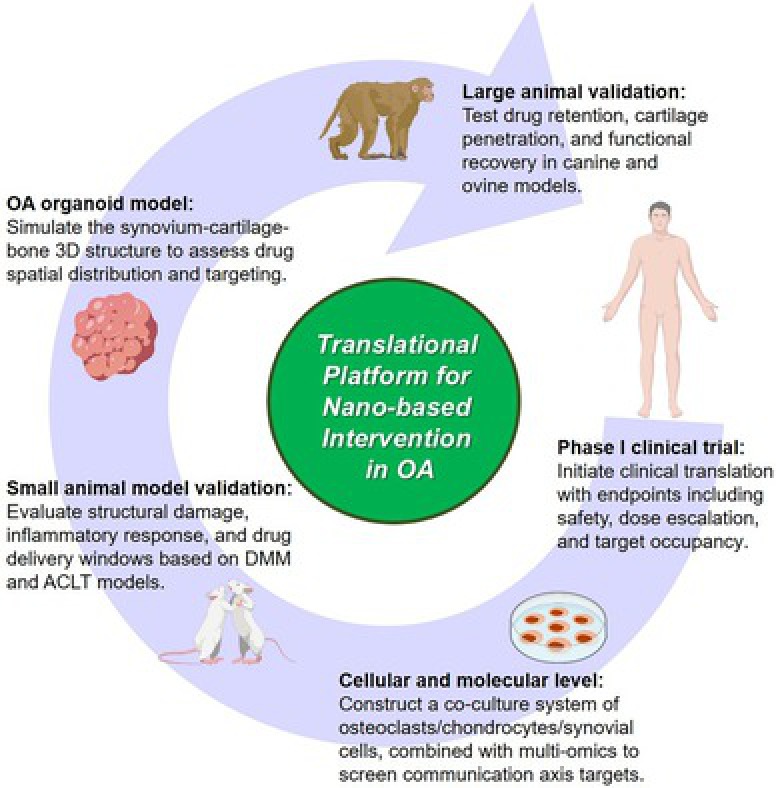

**Abbreviations**
ACLT: anterior cruciate ligament transection
AI: artificial intelligence
CatK: Cathepsin K
DMM: destabilization of the medial meniscus
DMOADs: disease‐modifying osteoarthritis drugs
EMA: European Medicines Agency
FDA: Food and Drug Administration
FLS: fibroblast‐like synoviocytes
GelMA: gelatin methacrylate
HIF‐2α: hypoxia‐inducible factor 2‐alpha
MRI: magnetic resonance imaging
MMP‐13: matrix metalloproteinase‐13
MMP‐9: matrix metalloproteinase‐9
NIR: near‐infrared
NF‐κB: nuclear factor kappa‐light‐chain‐enhancer of activated B cells
NSAIDs: non‐steroidal anti‐inflammatory drugs
OA: osteoarthritis
OPG: osteoprotegerin
PLGA: poly(lactic‐co‐glycolic acid)
RANK: receptor activator of nuclear factor κB
RANKL: receptor activator of nuclear factor κB ligand
RNAi: RNA interference
ROS: reactive oxygen species
scRNA‐seq: single‐cell RNA sequencing
TRAP: tartrate‐resistant acid phosphatase
VEGF: vascular endothelial growth factor

**Translational Impact Statement**

By elucidating pathological osteoclast–chondrocyte crosstalk as a central driver of osteoarthritis progression, this review highlights actionable molecular targets and advanced nanotherapeutic strategies with direct clinical relevance. Integrating small‐molecule inhibitors with targeted, stimuli‐responsive nanodelivery systems offers a feasible path to overcome current barriers in joint drug delivery, improve local efficacy, and reduce systemic toxicity. These insights provide a translational framework for developing disease‐modifying osteoarthritis therapies that bridge mechanistic discovery with precision, patient‐oriented clinical intervention.

## 1 INTRODUCTION

### 1.1 Current status and challenges of osteoarthritis (OA): From epidemiology to therapeutic bottlenecks

OA is a chronic joint disorder primarily characterized by degenerative changes in articular cartilage, accompanied by subchondral bone remodeling, synovial inflammation, and osteophyte formation.^1^ According to a Global Burden of Disease study published in *The Lancet Rheumatology* in 2023, OA affects more than 500 million people worldwide and has become one of the leading causes of mobility loss and reduced quality of life among middle‐aged and elderly populations.^2^ In the context of an aging society, both the incidence and prevalence of OA have shown a continuous upward trend.^3^, ^4^ In China, epidemiological surveys indicate that the prevalence of OA in individuals aged 60 years and older approaches 50%, with knee OA being the most common form.^5^ Beyond causing chronic pain and mobility impairment, OA imposes a substantial burden on healthcare systems and economic resources, especially given the rising demand for joint replacement surgeries, which has further intensified pressure on medical insurance systems.^6^, ^7^

**FIGURE 1:**
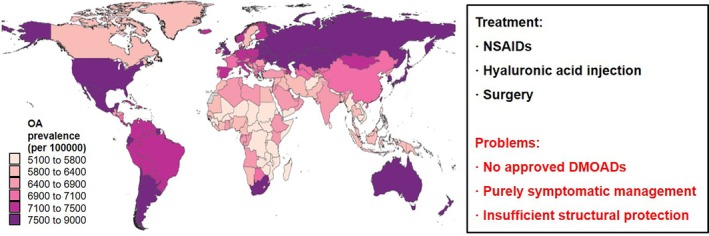
Global epidemiological trends and current treatment landscape of OA.

Despite the high rates of disability and economic burden associated with OA, no disease‐modifying osteoarthritis drugs (DMOADs) have been officially approved for clinical use by the U.S. Food and Drug Administration (FDA) or the European Medicines Agency (EMA) to date.^8^, ^9^ Current treatment strategies remain largely symptomatic and include oral non‐steroidal anti‐inflammatory drugs (NSAIDs), topical analgesics, intra‐articular injections (e.g., hyaluronic acid, corticosteroids), physical therapy, and joint replacement surgery.^10^, ^11^ Although these interventions can temporarily alleviate pain and improve mobility, they fail to reverse cartilage degeneration or halt the progression of bone structural changes. Clinical studies have shown that long‐term use of NSAIDs may result in adverse effects such as gastrointestinal ulcers and cardiovascular complications, while the efficacy of hyaluronic acid injections remains controversial and appears limited to certain patient subgroups.^12^, ^13^ Therefore, there is an urgent need in the OA field for disease‐modifying therapies capable of slowing disease progression and preserving joint structure (Figure 1).

### 1.2 Core pathology of OA: Structural damage and communication imbalance

**FIGURE 2:**
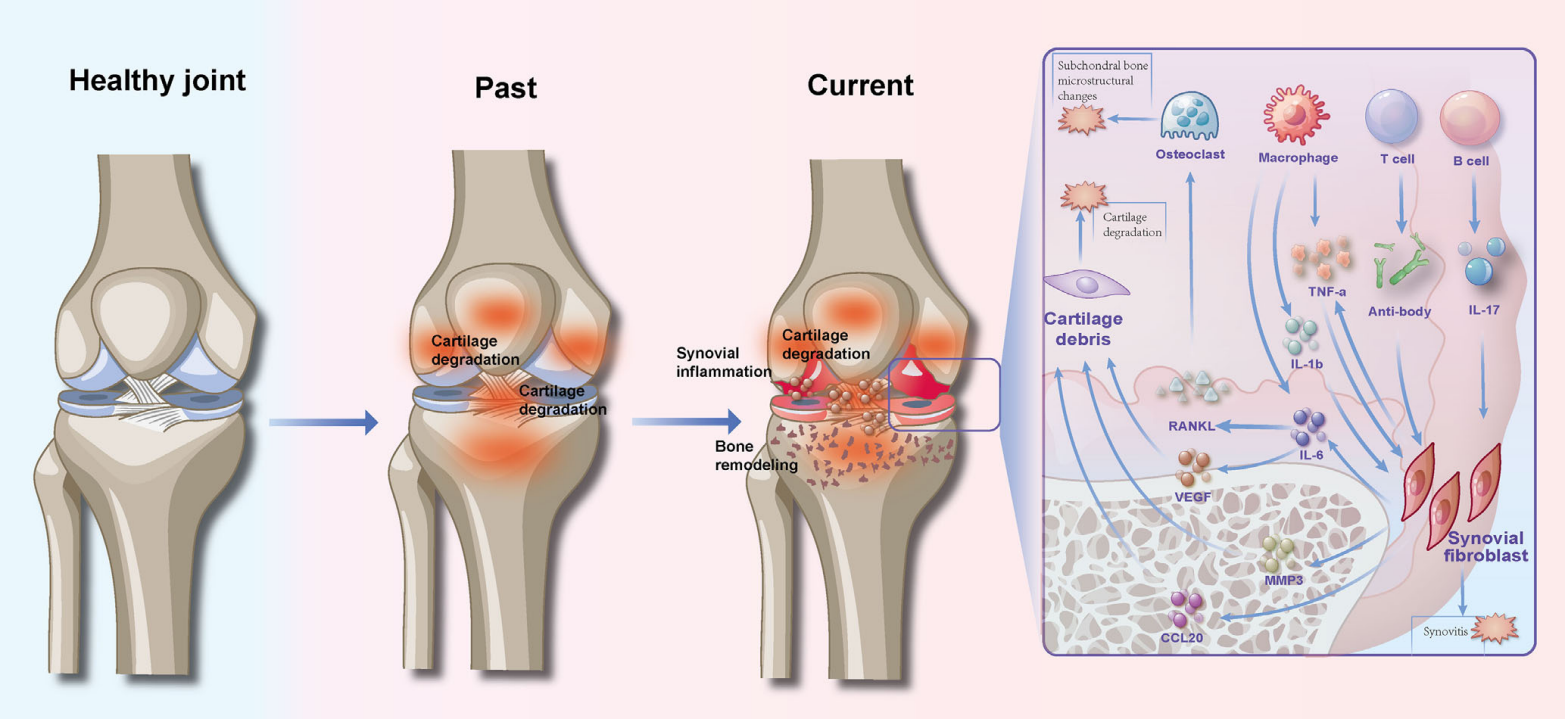
Conceptual evolution of OA from a “degenerative cartilage disease” to a “whole‐joint organ disorder”.

With increasing understanding of the pathological mechanisms of OA, the disease has been redefined as a systemic “whole‐joint organ disorder” rather than a condition limited to cartilage degeneration (Figure 2).^14^, ^15^ Magnetic resonance imaging (MRI) and histopathological studies have revealed that OA involves not only cartilage degradation but also microstructural changes in the subchondral bone plate, low‐grade synovial inflammation, meniscal damage, and osteophyte formation.^16^, ^17^ Among these, perforation and structural disruption of the subchondral bone plate are considered key initiating events in early‐stage OA. These changes activate osteoclasts, leading to bone resorption and localized inflammatory responses, thereby disturbing the homeostatic regulation between bone and cartilage.^18^

Throughout this process, intercellular signaling networks play a critical role. OA progression does not result from isolated abnormalities in a single cell type but is instead driven by a pathological communication axis formed through interactions among osteoclasts, chondrocytes, synovial fibroblast‐like cells, macrophages, and immune cells. These cells engage in crosstalk via cytokines, exosomes, chemokines, and other mediators.^19^ A study based on single‐cell RNA sequencing (scRNA‐seq) revealed a significant positive correlation between osteoclast activation and the expression level of matrix metalloproteinase‐13 (MMP‐13) in chondrocytes within OA joints, indicating a direct pathological coupling between the two cell types.^19^ The same study further demonstrated that, in the destabilization of the medial meniscus (DMM) model, osteoclast‐derived Cathepsin K (CatK) and exosomal miR‐21 could induce a degradative phenotypic shift in chondrocytes. This established a positive feedback loop of mutual activation and damage between osteoclasts and chondrocytes—one of the key mechanisms driving structural deterioration and inflammatory exacerbation in OA.^20^

### 1.3 From symptom management to mechanistic regulation: The evolution of targeted therapeutic strategies

Traditional treatments for OA have primarily focused on symptom relief and improving quality of life, but have failed to slow disease progression or restore joint structure. Multiple international clinical studies have confirmed the limitations of this approach. Evidence indicates that among all mainstream therapeutic options, only exercise therapy and certain intra‐articular injections have shown limited structural protective effects.^13^, ^21^, ^22^ Although surgical intervention can restore joint function, it is typically reserved for moderate to late‐stage OA and carries long‐term risks such as prosthesis wear and postoperative infection.^23^, ^24^ Consequently, the search for early‐stage interventions that can interrupt pathological progression has become a critical focus in OA research.

**FIGURE 3:**
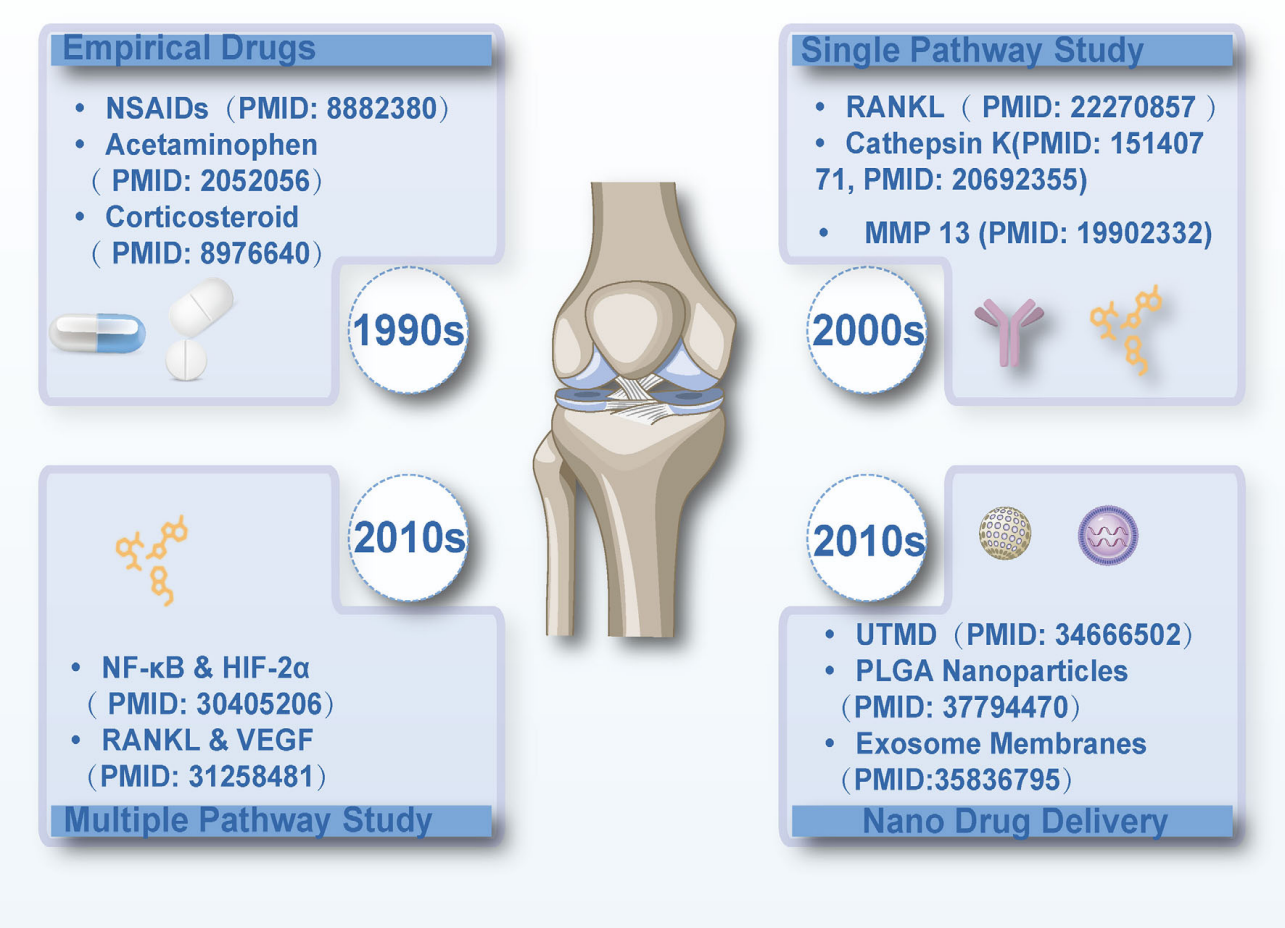
Transition of OA treatment strategies from symptomatic relief to mechanism‐based intervention.

In recent years, OA therapy has shifted from mere symptom management toward interventions targeting pathological mechanisms. Pathological signaling axes, such as the Receptor Activator of Nuclear Factor κB Ligand (RANKL)‐Receptor Activator of Nuclear Factor κB (RANK)/Osteoprotegerin (OPG) pathway, Nuclear Factor Kappa B (NF‐κB) signaling, Hypoxia‐Inducible Factor 2‐alpha (HIF‐2α), and Vascular Endothelial Growth Factor (VEGF), have been extensively studied and proposed as candidate targets for therapeutic intervention.^25^, ^26^ Small‐molecule drugs, antibody therapies, and RNA interference (RNAi) technologies targeting these pathways have demonstrated promising results in animal models. For example, inhibition of CatK effectively reduced osteoclast activity and bone resorption, thereby attenuating subchondral bone plate damage.^27^ As OA is increasingly recognized as a complex network‐driven disease involving multiple tissues and pathways, single‐target interventions have generally failed to achieve satisfactory efficacy in clinical trials. In recent years, the concept of multi‐pathway synergistic targeting has gained momentum, with several reviews suggesting that combination‐based, multi‐mechanism intervention strategies may overcome the therapeutic limitations of single‐target approaches (Figure 3).^28^, ^29^

### 1.4 Advances in small‐molecule inhibitors and nanodelivery systems

The development of small‐molecule drugs targeting osteoclast–chondrocyte communication mechanisms has progressed rapidly in recent years. Inhibitors of CatK (e.g., Odanacatib), MMP‐13, and NF‐κB (e.g., Bortezomib) have demonstrated significant efficacy in delaying bone resorption and cartilage degradation in animal models such as DMM and anterior cruciate ligament transection (ACLT).^30^ However, the therapeutic outcomes of these agents are often limited by the joint's physiological barriers, which result in poor tissue penetration and unstable local drug concentrations.^31^, ^32^ Moreover, systemic toxicity associated with long‐term systemic administration remains a major barrier to clinical translation. For example, although the MMP‐13 inhibitor PG‐116800 demonstrated certain biological activity in a clinical trial for knee OA, its development was terminated due to a high incidence of tendon‐related adverse effects.^33^

To overcome the limitations of drug delivery efficiency and targeting specificity, nanomedicine technologies have gradually been introduced into the field of OA therapy. Smart nanocarriers constructed from liposomes, poly(lactic‐co‐glycolic acid) (PLGA), metal oxides, and biomimetic materials have been designed to respond to local microenvironmental triggers—such as acidic pH, inflammatory enzymes, and reactive oxygen species (ROS)—to enable site‐specific drug release at the lesion site.^34^, ^35^ A study developed rapamycin‐loaded PLGA nanoparticles (RNPs) with high encapsulation efficiency and sustained‐release properties, which effectively prolonged the intra‐articular retention time of rapamycin. This formulation significantly alleviated cartilage destruction, osteophyte formation, chondrocyte hypertrophy, synovial inflammation, and pain.^36^ In addition, studies using OA animal models have explored dual‐drug delivery to articular cartilage via targeted carriers. For example, a WYRGRL‐modified nanocarrier (a collagen type II–targeting peptide) capable of simultaneously releasing two therapeutic agents was shown to delay cartilage degeneration in vivo.^37^ Nanoplatforms, which integrate drug loading, stimuli‐responsive release, and theranostic functionality, are increasingly emerging as key enabling technologies for mechanism‐oriented OA therapeutic strategies.^38^, ^39^

This review aims to systematically summarize the pathological roles of osteoclast–chondrocyte communication in OA and evaluate its potential as a therapeutic target. It also outlines the current research status and mechanistic features of small‐molecule inhibitors that interfere with communication axes, explores how nanodelivery systems can be harnessed for precise modulation of these networks, and analyzes the key challenges and future directions for clinical translation.

## 2 OA JOINT ECOLOGY AND OSTEOCLAST‐CARTILAGE COMMUNICATION MECHANISMS: FROM HOMEOSTASIS TO PATHOLOGICAL ACTIVATION

### 2.1 Multicellular composition and the complex microenvironment of the OA joint

The OA joint is not composed of a single tissue but comprises multiple structural components, including cartilage, subchondral bone, synovium, synovial fluid, meniscus, and ligaments. It involves various cell types such as chondrocytes, osteoclasts, fibroblast‐like synoviocytes (FLS), synovial macrophages, osteoblasts, osteocytes, and immune cells, including T cells, B cells, macrophages, and natural killer cells.^40^, ^41^ Notably, within bone tissue, osteoblasts and osteocytes play central roles in bone remodeling and the maintenance of homeostasis. These cells form a highly complex signaling network through the secretion of cytokines, chemokines, exosomes, and matrix‐degrading enzymes, collectively maintaining the homeostasis of the joint microenvironment.^42^, ^43^, ^44^ As OA progresses, the multicellular network that maintains joint homeostasis becomes increasingly disrupted, characterized by synovial infiltration of macrophages and lymphocytes and synovial hyperplasia, accompanied by osteoclast activation and an imbalance in the remodeling of the subchondral bone microenvironment. Osteoclast activation and cartilage degradation proceed in a coordinated manner.^45^

scRNA‐seq has revealed the dynamic evolution of cellular subpopulations throughout OA development. Studies have shown that within the OA synovium, FLS adopt a pro‐inflammatory phenotype characterized by the expression of IL‐6 and MMP3, accompanied by an upregulation of CD86^+^ macrophages, collectively amplifying local inflammatory signaling.^46^ Additionally, a newly identified subset of “fibrocartilage‐like chondrocytes” has emerged within the chondrocyte lineage. These cells express high levels of COL1A1, RUNX2, and VEGF and are closely associated with osteogenic transdifferentiation.^47^ In the osteoclast lineage, abnormally activated subpopulations characterized by elevated tartrate‐resistant acid phosphatase (TRAP) expression have also been identified, indicating a pathological expansion of osteoclast‐mediated bone remodeling in OA.^47^ Dynamic profiling of these subpopulations offers the potential to reconstruct the heterogeneous histopathological landscape of the OA joint and provides a cellular basis for precision‐targeted therapies.^48^ The spatial and temporal characteristics of these cell lineages have been validated through immunofluorescence staining of synovial tissues in OA animal models and single‐cell spatial transcriptomics in human OA joint tissues, confirming their stable and region‐specific features during disease progression. These findings highlight their potential as biological foundations for drug targeting and disease subtyping.^49^, ^50^, ^51^

### 2.2 Regulatory mechanisms of bone‐cartilage coupling under normal conditions

In healthy joints, chondrocytes and the subchondral bone are connected via the cartilage endplate and calcified layer. Although anatomically separated, they remain metabolically and functionally coupled during development, maintenance, and tissue repair. The endplate cartilage facilitates nutrient and signaling exchange with the underlying trabecular bone through small channels, a process that is especially crucial during embryonic ossification and postnatal bone remodeling.^52^, ^53^ Chondrocytes contribute to bone formation and inhibit bone resorption through pathways such as Wnt, TGF‐β, and BMP, while osteoclasts, in turn, regulate cartilage structure by modulating endplate porosity and osteogenic signaling.^28^, ^54^ This coupling system maintains the microenvironmental stability of joint architecture and is essential for preserving joint tissue functionality.^55^

**FIGURE 4:**
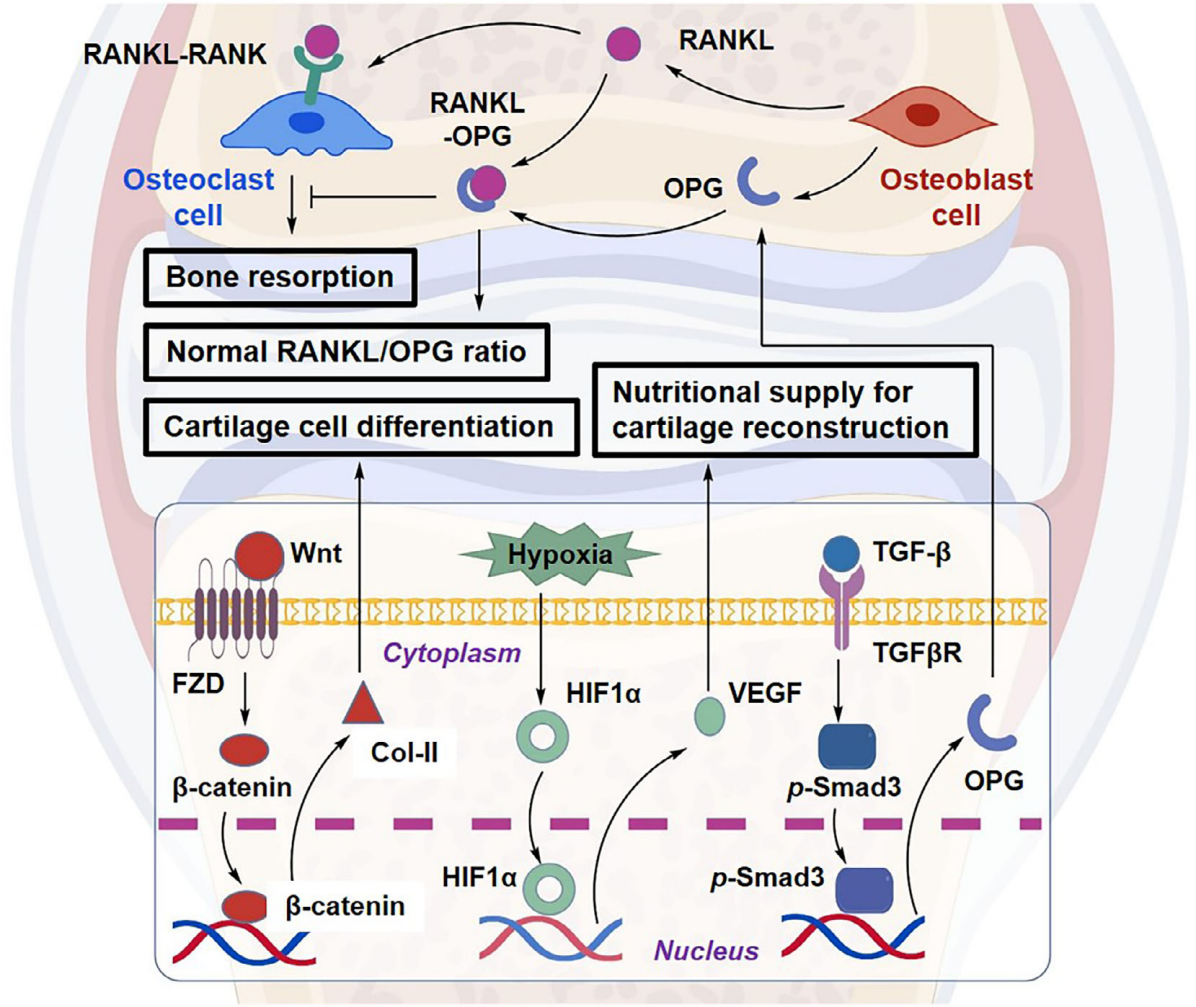
Diagram of osteochondral architecture and coupling signaling networks in healthy joints.

Several signaling axes work in concert to sustain bone‐cartilage homeostasis. The RANKL/OPG axis maintains the balance of bone resorption; Wnt/β‐catenin regulates the differentiation trajectory of chondrocytes; TGF‐β signaling exerts dual regulatory effects on subchondral bone plate remodeling; and VEGF, as a key regulator of local angiogenesis, indirectly participates in the transport of nutrients within cartilage tissue by modulating the number and function of vascular buds in subchondral bone (such as vertebral endplate osseous regions), while directly influencing the extent and efficiency of bone remodeling.^56^, ^57^ Animal studies support the role of the RANKL/OPG gradient in maintaining cartilage–bone marrow homeostasis. External perturbations such as VEGF and TGF‐β can disrupt this balance through inflammatory mechanisms or aberrant signaling, leading to pathological changes characteristic of osteoarthritis.^51^, ^58^, ^59^ For example, in VEGF‐overexpressing mice, endplate perforation was observed to occur concurrently with upregulation of RANKL, whereas treatment with a TGF‐β receptor inhibitor significantly alleviated the uncoupling between osteoclastic activity and cartilage structure. These findings suggest that dysregulation of this signaling axis may represent an early event in OA and may provide a potential therapeutic intervention window (Figure 4).^60^

### 2.3 Pathological activation of the osteoclast–chondrocyte communication axis and the “mutual activation–co‐damage” loop

Abnormal bidirectional communication between osteoclasts and chondrocytes constitutes a core pathological mechanism driving the progression of OA. In the early stages of OA, damaged chondrocytes, under mechanical stress and inflammatory stimulation, release molecules such as RANKL and IL‐1β that traverse the bone–cartilage interface, activating osteoclast precursors in the subchondral bone and promoting their differentiation and activation.^20^ Conversely, activated osteoclasts exacerbate cartilage destruction through multiple mechanisms: (1) by secreting cathepsin K, which directly degrades the subchondral bone matrix, compromising its supportive structure and potentially affecting the overlying cartilage; (2) by releasing ROS, which intensify local oxidative stress; and (3) by delivering functional RNAs such as miR‐21 via extracellular vesicles or exosomes into chondrocytes, thereby modulating gene expression, upregulating matrix‐degrading enzymes such as MMP‐13, and promoting the phenotypic shift of chondrocytes toward hypertrophic or catabolic states.^47^

**FIGURE 5:**
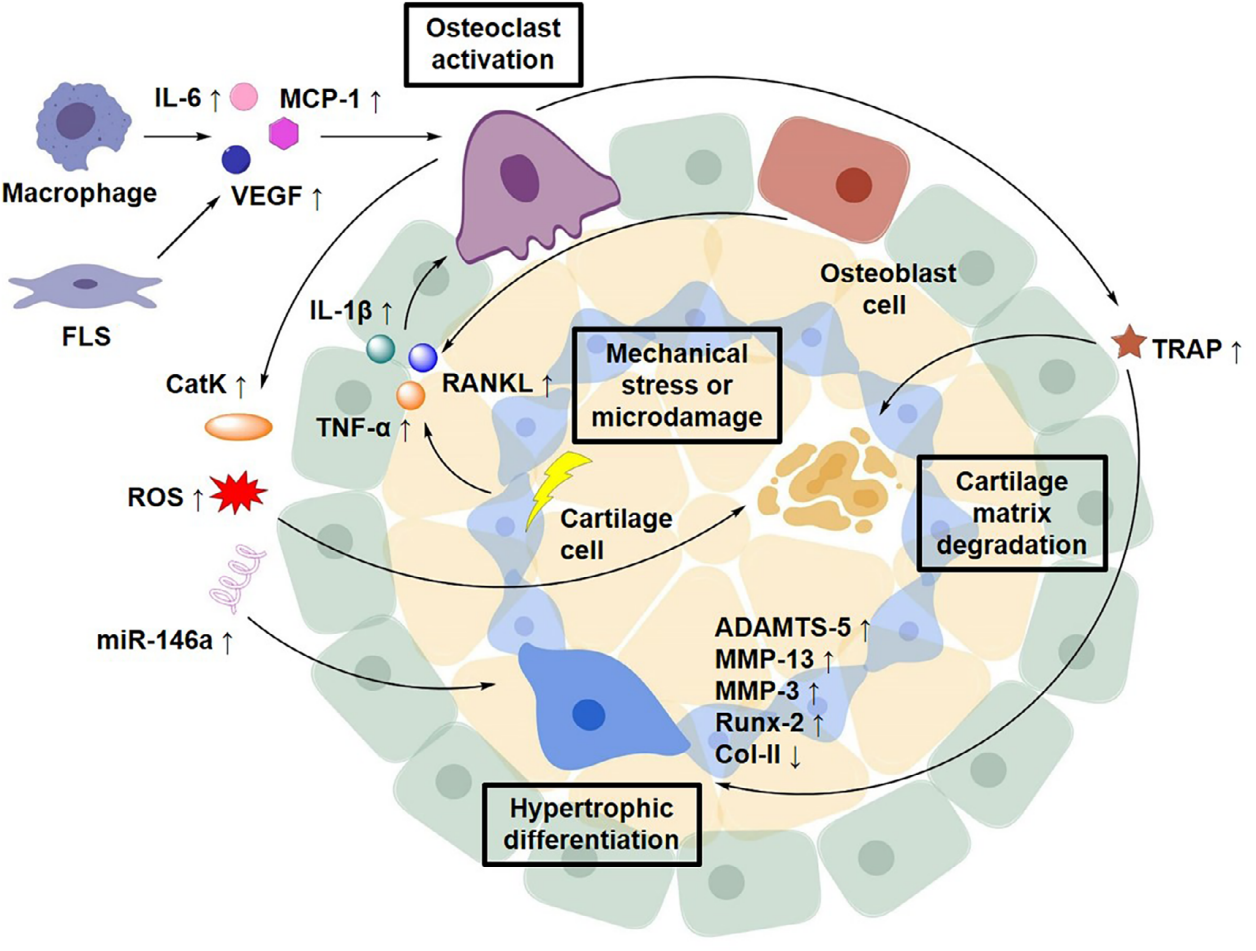
Feedback loop between osteoclasts and chondrocytes under OA conditions.

This process establishes a self‐reinforcing positive feedback loop—signals from chondrocytes activate osteoclasts, while osteoclast‐derived feedback further amplifies chondrocyte catabolism—forming a “mutual activation–co‐damage” circuit. Temporal analyses in the DMM mouse model have confirmed this loop, characterized by synchronized upregulation of cathepsin K and MMP‐13 expression, alongside the concurrent aggravation of bone resorption and cartilage erosion (Figure 5).^61^ Moreover, macrophages and fibroblast‐like synoviocytes (FLS) within the synovium amplify this pathological communication axis by secreting pro‐inflammatory cytokines such as IL‐6 and MCP‐1.^62^, ^63^

### 2.4 Core signaling pathways and therapeutic targets of the osteoclast–chondrocyte communication axis

**FIGURE 6:**
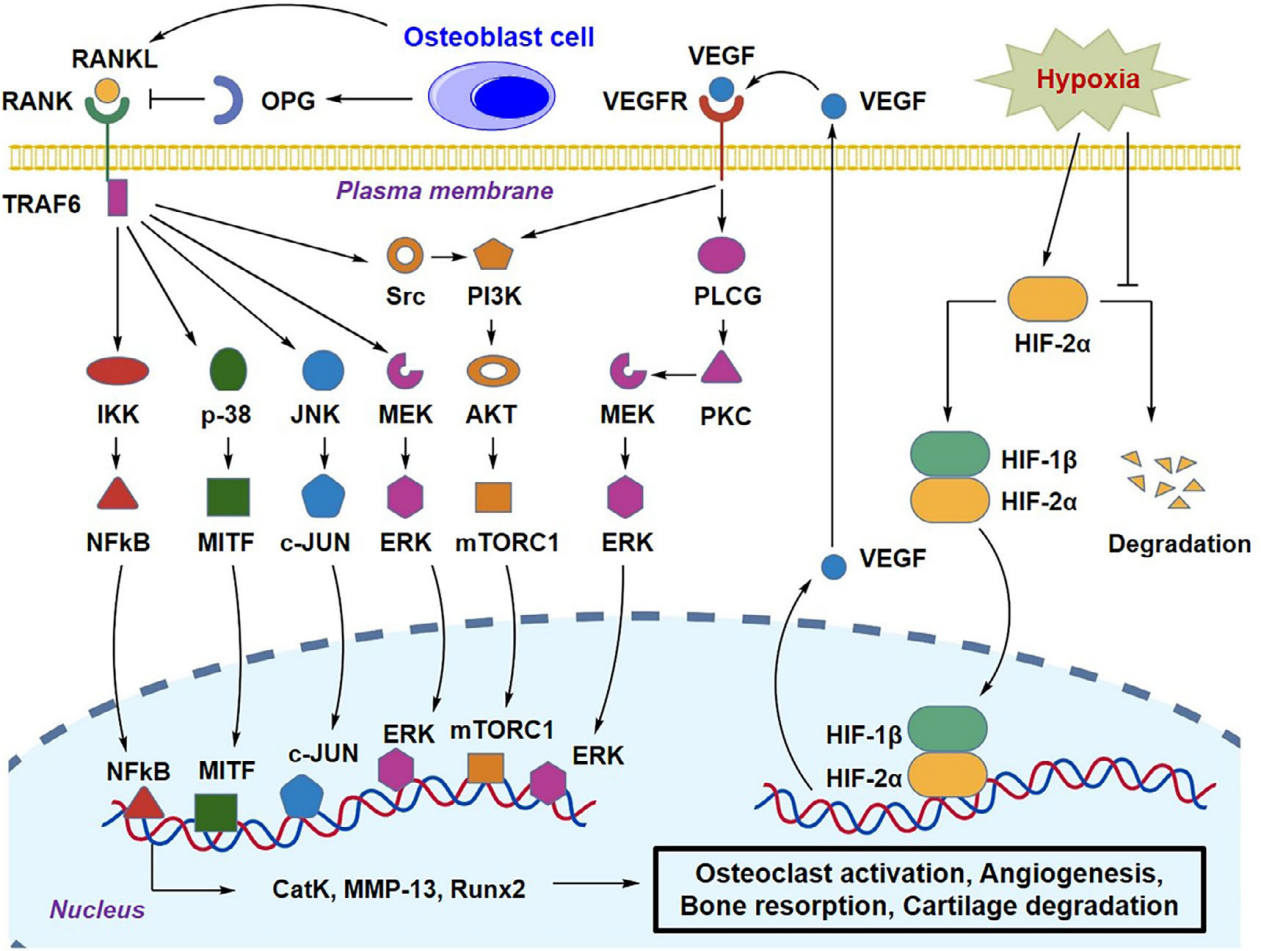
Integrated map of key signaling pathways involved in the osteoclast–chondrocyte communication axis.

The osteoclast–chondrocyte communication axis in OA involves the synergistic activation of multiple signaling pathways, which together drive the bidirectional imbalance between bone resorption and cartilage degradation (Figure 6). Among these, the RANKL–RANK/OPG axis serves as the central regulator of bone metabolic homeostasis. In OA, the equilibrium of this system is disrupted, with widespread upregulation of RANKL expression. Notably, osteoprogenitor cells located on bone surfaces are the principal physiological source of membrane‐bound RANKL, whereas osteocytes and mature osteoblasts mainly produce the decoy receptor OPG. Dysregulation of this spatially defined cellular division of labor is a key contributor to pathological bone resorption.^64^ In addition to RANKL signaling, hypoxia‐inducible factor HIF‐2α is stably expressed in OA chondrocytes and functions as a key upstream transcription factor that directly drives the transcription of major catabolic enzymes such as MMP‐13 and ADAMTS‐5, thereby promoting cartilage matrix degradation.^65^ Vascular endothelial growth factor (VEGF) not only facilitates aberrant vascular invasion into the subchondral bone but also directly participates in bone remodeling, with its overexpression closely associated with structural damage such as endplate perforation.^60^ Moreover, NF‐κB can be activated by multiple stimuli, including IL‐1β and TNF‐α, subsequently upregulating osteoclastogenesis and the expression of various catabolic enzymes in chondrocytes. It serves as a central node in inflammatory amplification and phenotype transition.^28^, ^66^, ^67^ These pathways do not operate in isolation but rather form an integrated regulatory network that sustains abnormal activity within the osteoclast–cartilage communication axis. The subchondral bone microenvironment, through RANKL–NF‐κB and TGF‐β signaling, further drives cartilage degeneration, providing a mechanistic basis for the development of small‐molecule and nanotechnology‐based targeted intervention strategies.^28^, ^45^

The subsequent sections of this review will systematically summarize therapeutic targets associated with these signaling hubs, covering their regulatory mechanisms, the current status of small‐molecule inhibitor development, and validation in preclinical models. The goal is to provide both a theoretical framework and practical guidance for the precise modulation of these communication networks. It is worth noting that although the figures and discussions in this review primarily focus on knee OA—the most extensively studied and best‐modeled form—the aberrant communication between osteoclasts and chondrocytes is considered a shared pathological basis across different joint sites. However, OA affecting various joints differs in etiology, progression rate, and the extent of tissue involvement. For instance, hand OA exhibits a stronger genetic predisposition and is often associated with metabolic syndrome. Its pathological progression is characterized by pronounced inflammatory responses in chondrocytes and prominent osteophyte formation, such as Heberden's nodes.^68^, ^69^ In contrast, hip OA is more closely linked to biomechanical factors, such as femoroacetabular impingement, and displays marked subchondral bone remodeling and sclerosis even in the early stages of disease progression.^70^, ^71^ Spinal OA, involving facet joints and intervertebral discs, is closely associated with vertebral endplate bone remodeling and neural compression.^72^, ^73^ Despite these regional distinctions, core signaling pathways—including the RANKL/RANK/OPG axis, NF‐κB, and HIF‐2α—have been consistently shown to be activated across different joint types, suggesting that therapeutic strategies targeting the osteoclast–chondrocyte communication axis may have broad applicability.^74^, ^75^ Therefore, although the mechanisms and nanotherapeutic strategies summarized in this review are discussed mainly within the context of the knee joint, the underlying principles provide a generalizable framework for the development of disease‐modifying therapies applicable to OA affecting multiple joint sites.

## 3 IDENTIFICATION OF COMMUNICATION TARGETS AND ADVANCES IN SMALL‐MOLECULE INTERVENTIONS

Building upon the key signaling pathways of the aforementioned communication axes, recent studies have proposed several small‐molecule intervention targets that aim to simultaneously regulate osteoclast and chondrocyte activity. The following sections provide a focused discussion of these targets and the experimental basis supporting their therapeutic potential.

### 3.1 Key mediators of bone resorption and cartilage degradation

#### 3.1.1 Evidence for the expression and mechanistic roles of RANKL, CatK, and MMP‐13 in OA

RANKL is the central factor governing osteoclast differentiation. Within the bone microenvironment, its expression exhibits a distinct spatial distribution: osteoprogenitor cells located on the bone surface are the primary source of membrane‐bound RANKL, which activates osteoclasts, whereas osteocytes and osteoblasts mainly produce OPG. Together, these factors finely regulate bone resorption.^59^, ^64^, ^76^, ^77^ In the subchondral bone of OA, this homeostatic balance is disrupted: RANKL expression is markedly upregulated in both bone surface cells and osteocytes, driving aberrant bone remodeling. Meanwhile, the compensatory coupling mechanism, in which osteoclasts secrete vRANK to activate reverse RANKL signaling and promote bone formation, also becomes dysregulated.^59^, ^78^, ^79^, ^80^ Furthermore, under inflammatory stimulation, chondrocytes, FLS, and T cells become additional pathological sources of RANKL. T cells initiate the early inflammatory response, while activated FLS and chondrocytes—acting as resident tissue cells—not only respond to inflammatory signaling but also directly promote osteoclast differentiation through RANKL secretion. Factors released during bone resorption (such as calcium ions) further stimulate immune and stromal cells, creating a self‐amplifying pathological cascade that exacerbates the vicious cycle of osteoclast activation.^41^, ^81^, ^82^, ^83^ CatK is a collagen‐degrading protease secreted specifically by osteoclasts and is critical for the resorption of type I collagen in the subchondral bone matrix. Studies using animal models have shown that CatK expression increases significantly as OA progresses in the DMM model and that its local activity closely correlates with cartilage thinning.^41^ Additionally, osteoclast‐derived ROS and extracellular vesicles also contribute to modulating the inflammatory microenvironment and the activity of the cell communication axis at the bone‐cartilage interface.

**FIGURE 7:**
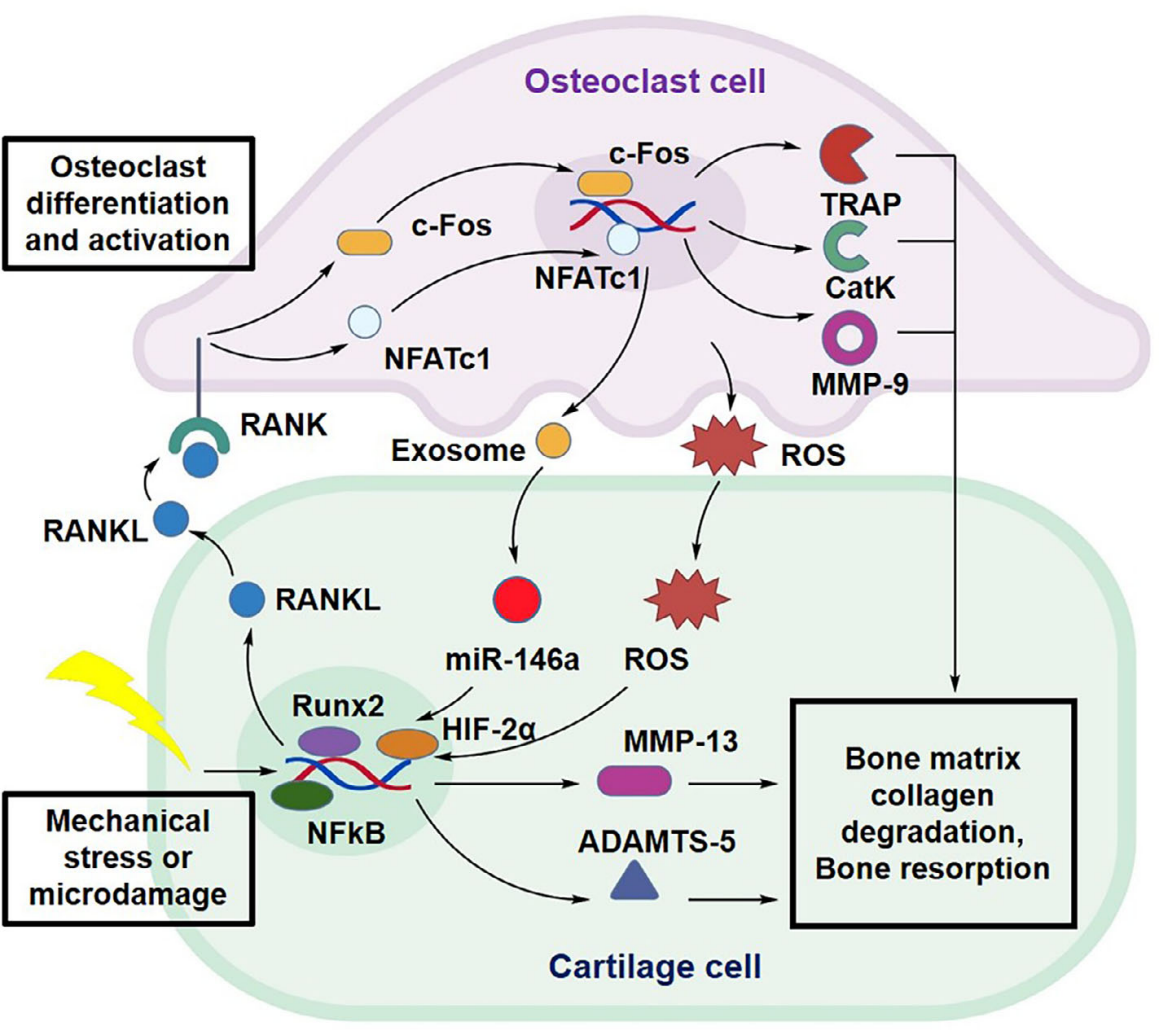
Core molecular mechanisms of bone resorption and cartilage degradation in OA, including target distribution.

In chondrocytes, the MMP family—particularly MMP‐13—is closely associated with cartilage matrix degradation. MMP‐13 cleaves type II collagen and is one of the earliest and most potent enzymes activated in OA. ADAMTS‐5, a key aggrecanase, degrades proteoglycans by cleaving aggrecan, the major proteoglycan in cartilage. The transcription factors HIF‐2α, Runx2, and NF‐κB act as upstream regulators of these catabolic enzymes. Studies have shown that HIF‐2α expression is significantly elevated in OA cartilage, accompanied by the upregulation of MMP‐13 and ADAMTS‐5. Cartilage‐specific deletion of HIF‐2α suppressed the expression of these enzymes and delayed OA progression.^65^ Moreover, Runx2 not only regulates chondrocyte maturation and mineralization but also synergistically promotes MMP‐13 expression, serving as a critical driver of the phenotypic transition from cartilage to hypertrophic or osteogenic states.^41^ Together, these factors constitute a core pathogenic network linking bone resorption and cartilage degradation in OA and offer multiple potential molecular targets for small‐molecule intervention (Figure 7).

The expression and activation of these molecules were validated in osteoclast induction systems. Following RANKL stimulation, the expression of TRAP and CatK was significantly upregulated and positively correlated with functional indices of osteoclast activity.^84^ Immunohistochemical analyses of mouse OA models further confirmed that CatK was predominantly localized to subchondral bone resorption zones,^85^ underscoring its essential role in pathological bone remodeling.

### 3.2 Advances in small‐molecule therapeutics targeting bone resorption and cartilage preservation

#### 3.2.1 Small‐Molecule Inhibitors Targeting Bone Resorption–Related Pathways

The process of bone resorption is regulated by signaling pathways associated with osteoclast activation, proliferation, and functional execution. Currently developed small‐molecule inhibitors primarily target key osteoclastic signaling hubs and essential effector proteins, as detailed below:

CatK Pathway Inhibitors: Cathepsin K (CatK) is a core therapeutic target for bone resorption intervention. Odanacatib, a representative inhibitor, has progressed to phase III clinical trials. In animal models, Odanacatib significantly suppresses osteoclastic resorptive activity, reduces trabecular bone loss, and improves the microstructural integrity of subchondral bone, thereby laying the groundwork for clinical translation.^86^, ^87^

Src/NFATc1 Signaling Axis Inhibitors: NFATc1 and c‐Src kinase represent central signaling hubs governing osteoclast differentiation and function. Bafetinib, a dual c‐Src/ABL inhibitor, has demonstrated clear efficacy in ACLT animal models by reducing osteoclast numbers through inhibition of c‐Src activity, alleviating trabecular bone rarefaction, and delaying structural bone damage.^88^

RANKL–RANK Pathway Inhibitors: The RANKL–RANK axis provides the key initiating signal for osteoclast activation. Among its inhibitors, Denosumab—a monoclonal antibody—has been approved for the treatment of osteoporosis. However, its systemic immunosuppressive effects pose potential risks to the homeostasis of the local joint immune microenvironment, limiting its current application in OA therapy to the preclinical stage.^89^ To date, no small‐molecule inhibitors targeting the RANKL–RANK pathway have been approved by regulatory authorities such as the FDA or EMA, nor have any entered phase III clinical trials.

Collectively, the above‐mentioned small‐molecule inhibitors targeting bone resorption pathways have undergone pharmacokinetic and pharmacodynamic evaluations in animal models, demonstrating potent bone‐protective effects and structural stability without inducing significant systemic toxicity. These findings indicate a solid preclinical foundation for their further development as therapeutic agents for bone resorption intervention (Table 1).

**TABLE 1:** Overview of developed small‐molecule inhibitors and their molecular targets.

Compound	Odanacatib	AZD0530	Denosumab	CL82198	UK‐356618	PT2385	CADD522	BAY11‐7082	Parthenolide
Structure	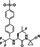	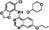	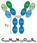	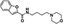	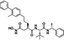	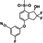	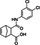	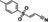	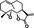
Target	CatK	c‐Src/ABL	RANKL	MMP‐13	MMP‐3	HIF‐2α	Runx2	NFkB	NFkB/HDAC1
Mechanism	Inhibitor	Inhibitor	Antibody	Inhibitor	Inhibitor	Inhibitor	Inhibitor	Inhibitor	Inhibitor
Preclinical model	DMM/ACLT‐induced OA	DMM‐induced OA	DMM‐induced OA	MLI‐induced OA	–	–	–	–	–
Comments	Bone resorption ↓ Osteophyte formation↑	cartilage destruction ↓ subchondral bone sclerosis↓	Osteoclast differentiation, activation ↓ Bone resorption ↓	MMP13 activity ↓ Col‐II and proteoglycan↑	MMP‐3 IC_50_ = 5.9 nM (>140‐fold less potent against MMP‐1/2/9/14)	HIF2α‐ARNT↓ (K_i_ < 50 nM) MMPs ↓	RUNX2‐DNA ↓ (IC_50_ = 10 nM) ROS ↓ MMP‐13 ↓	p‐IκBα ↓ USP7_IC_50_ = 0.19 μM, USP21_IC_50_= 0.96 μM	NF‐κB ↓ HDAC1 ↓
Reference	PMID: 30773420	PMID: 29555825	PMID: 38361122, PMID: 27641143	PMID: 23298463	PMID: 35936461	PMID: 39321898	PMID: 39542509	PMID: 30316071	PMID: 25903444

#### 3.2.2 Small‐molecule inhibitors targeting cartilage‐protective pathways

The central goal of cartilage protection is to inhibit extracellular matrix (ECM) degradation and maintain the normal differentiation phenotype of chondrocytes. Current small‐molecule inhibitors in this field primarily target matrix‐degrading enzymes, inflammatory signaling pathways, and chondrocyte differentiation regulators, as outlined below:

Matrix‐Degrading Enzyme Inhibitors: Key enzymes responsible for degrading type II collagen in cartilage include MMP‐13 and ADAMTS‐5. MMP‐13 inhibitors such as CL82198 and UK‐356618 have been shown in OA animal models to markedly suppress cartilage matrix degradation, reducing the loss of type II collagen and proteoglycans and thereby delaying OA‐associated structural damage.^90^, ^91^ Additionally, ADAMTS‐5 inhibitors are under active investigation, though preclinical data remain inconclusive.^92^

HIF‐2α Signaling Pathway Inhibitors: HIF‐2α promotes cartilage degradation by forming a complex with ARNT to activate downstream MMPs (e.g., MMP‐13). The small‐molecule inhibitor PT2385 specifically blocks the interaction between HIF‐2α and ARNT, thereby downregulating MMP transcription. In vitro studies using chondrocytes have demonstrated the protective effects of PT2385 on the cartilage matrix.^93^

Runx2 Pathway Inhibitors: Runx2 is a key transcription factor regulating chondrocyte hypertrophic differentiation. Its overactivation leads to phenotypic abnormalities and accelerated cartilage senescence. The small molecule CADD522 selectively binds to the DNA‐binding domain of Runx2, inhibiting Runx2‐mediated MMP13 expression while maintaining the normal differentiation phenotype of chondrocytes, thus ameliorating cartilage abnormalities.^94^, ^95^

NF‐κB Inflammatory Signaling Pathway Inhibitors: The NF‐κB pathway is a central amplifier of local joint inflammation in OA, promoting cartilage degradation by upregulating pro‐inflammatory cytokines such as IL‐1β and TNF‐α. Representative inhibitors include BAY11‐7082, Parthenolide (a natural sesquiterpene lactone from *Tanacetum parthenium*), and IKKβ‐specific inhibitors, which block NF‐κB nuclear translocation or kinase activation, thereby attenuating inflammatory signaling and reducing ECM degradation.^96^, ^97^

Notably, the mechanisms of CADD522 and PT2385 have been validated in in vitro chondrocyte cultures and OA organoid models. Post‐intervention, both compounds significantly downregulate MMP‐13 and ADAMTS‐5 protein expression levels and suppress chondrocyte hypertrophic transition, indicating that these small molecules possess a stable molecular basis for modulating cartilage differentiation.

In summary, current small‐molecule inhibitors collectively target multiple levels of OA pathogenesis—ranging from initiating and effector signals of bone resorption to enzymatic degradation, differentiation regulation, and inflammatory amplification in cartilage protection—thereby offering substantial potential for combinatorial therapeutic strategies. However, challenges remain: early‐generation enzyme inhibitors (e.g., MMP‐13 inhibitors) often suffer from limited target selectivity, off‐target effects, and dose‐dependent toxicity. Future development should focus on structure‐based optimization (such as introducing specific functional groups) and targeted delivery strategies (e.g., nanoparticle encapsulation) to enhance therapeutic specificity and clinical safety.

### 3.3 Small‐molecule inhibitor‐based interventions: advantages, limitations, and mechanistic validation

#### 3.3.1 Advantages of small‐molecule inhibitor‐based intervention strategies

Compared with protein‐based antibody therapeutics or cell‐based therapies, small‐molecule inhibitors possess several inherent advantages. Owing to their low molecular weight (typically <1000 Da), stable chemical synthesis, and strong tissue permeability, they are suitable for both oral administration and intra‐articular injection, offering favorable bioavailability within the body through passive diffusion or carrier‐mediated delivery.^86^, ^98^ The molecular targets of these inhibitors are most often enzyme catalytic pockets or ligand‐binding domains of receptors, allowing their mechanisms of action to be clearly validated via molecular docking and in vitro enzyme activity assays. This enables precise “target–pharmacodynamic” correlations, which are critical for rational drug design. Representative examples include Odanacatib and SR9238, both of which have undergone comprehensive pharmacokinetic and toxicological evaluations. These studies revealed no apparent dose‐limiting toxicity, highlighting their promising potential for clinical translation.^86^

#### 3.3.2 Bottlenecks of small‐molecule inhibitor‐based intervention strategies

In the treatment of OA, small‐molecule drugs face substantial delivery challenges. Due to the dense barrier properties of cartilage and subchondral bone, along with the continuous dilution and metabolic clearance effects of synovial fluid, free drugs struggle to maintain therapeutic concentrations within the joint cavity. Fluorescence‐tracing studies in animal models have shown that within 24 h after intra‐articular injection, most free drugs are rapidly cleared, and local residual concentrations often fall below therapeutic thresholds, significantly compromising efficacy.^99^, ^100^

To overcome these delivery barriers, researchers have developed functionalized nanoplatforms that integrate small‐molecule inhibitors into targeted and stimulus‐responsive drug delivery systems. In a representative study, researchers successfully engineered PLGA‐based nanoparticles loaded with rapamycin (RNPs). These nanoparticles exhibited high encapsulation efficiency and sustained‐release properties, effectively prolonging the intra‐articular residence time of rapamycin. In mice subjected to DMM, once‐weekly intra‐articular administration of RNPs significantly attenuated articular cartilage destruction, inhibited osteophyte formation and chondrocyte hypertrophy, reduced synovial inflammation, and effectively improved pain‐related behaviors.^36^ This “small‐molecule drug + functional nanocarrier” synergistic approach represents a promising technological route to enhance the local efficacy of OA therapeutics.

Given that OA progression involves intersecting pathological cascades, single‐target inhibitors often fail to produce sustained disease‐modifying outcomes. Consequently, multi‐target combination inhibition strategies have gained increasing attention in recent years. Typical preclinical combinations include: 1, CatK inhibitor + MMP‐13 inhibitor, 2, NF‐κB inhibitor + HIF‐2α antagonist, and 3, Runx2 inhibitor + RANKL–RANK pathway inhibitor. Studies using DMM and ACLT animal models have demonstrated that these combination therapies produce synergistic effects, simultaneously suppressing bone resorption and cartilage degradation while improving local joint inflammation (evidenced by reduced IL‐1β and TNF‐α levels), cartilage surface integrity (lower OARSI scores), and osteophyte formation. Their efficacy notably surpasses that of monotherapies.^93^ For instance, a combination of HIF‐2α gene knockout and CatK inhibition enhanced cartilage protection by approximately 2.2‐fold compared with CatK inhibition alone, further validating the feasibility of dual‐pathway synergistic modulation.^93^

#### 3.3.3 Mechanistic validation pathways for small‐molecule inhibitor‐based interventions

In addition to pharmacological advances, mechanistic validation and efficacy evaluation platforms for OA therapeutics are also undergoing rapid evolution. Traditional small‐animal models, while useful for rapid preliminary assessment of drug efficacy, exhibit substantial limitations in clinical translatability due to pronounced differences in joint anatomy, immune background, and metabolic profile compared with humans. Recently, the development of human‐derived OA organoid models incorporating a three‐layer structure of synovium–cartilage–bone has emerged as a promising technological direction. These models are established by co‐culturing synoviocytes, chondrocytes, and osteoblasts derived from OA patients, thereby recapitulating in vivo–like intercellular communication, drug delivery barriers, and inflammatory microenvironments. When combined with advanced analytical techniques—such as real‐time fluorescence imaging, immunohistochemistry, and transcriptomic sequencing—these systems enable comprehensive evaluation of dose–response relationships, drug‐release kinetics, and signaling‐pathway regulation patterns, providing a preclinical testing framework that is more physiologically relevant to human disease.^101^, ^102^

Looking forward, an integrated multi‐scale research framework that combines small‐animal models, organoid systems, and large‐animal validation holds significant potential to establish a closed‐loop strategy spanning from target identification and mechanistic exploration to efficacy evaluation and drug‐delivery system design. Such an approach is expected to accelerate the clinical translation of personalized, multi‐target OA therapeutic interventions (Figure S1).^28^, ^103^, ^104^

## 4 NANODELIVERY STRATEGIES FOR PRECISION MODULATION OF COMMUNICATION AXES

As OA treatment paradigms shift from symptom relief to targeted modulation of pathological mechanisms, nanodelivery systems have emerged as a key tool to overcome current therapeutic limitations due to their superior targeting capacity, formulation stability, and programmable release features. This section systematically outlines the development trajectory of nanodelivery strategies in OA across five domains: carrier type, functional modification, stimuli‐responsive release, dual‐targeting synergy, and smart delivery systems.

### 4.1 Nanocarrier types and fundamental properties

Nanodelivery systems, owing to their small particle size, large surface area, tunable release profiles, and modifiable surfaces, have become critical tools for overcoming anatomical barriers and enhancing local bioavailability in OA treatment.^105^, ^106^ The distinct morphologies, intra‐lesional distribution patterns, and therapeutic outcomes of these diverse nanocarriers are summarized in Table S1. Compared with conventional intra‐articular drug injections, nanocarriers significantly improve drug stability in the synovial environment, enhance accumulation at target cells or tissues, and prolong intra‐articular retention time. In OA nanotherapy, local intra‐articular injection currently represents the most mainstream delivery strategy. Compared with systemic administration, it can markedly enhance joint bioavailability and reduce systemic toxicity. However, major challenges remain, including rapid drug clearance by synovial fluid and the limited penetration of dense cartilage ECM.^107^ Nanodelivery systems offer effective solutions to these obstacles by prolonging intra‐articular drug retention, enhancing ECM permeability, and achieving targeted delivery, thereby maximizing both residence time and targeting efficiency within the joint.^108^ In addition, tissue‐implantable delivery systems have been developed to provide long‐term and controlled drug release, making them particularly suitable for chronic disease management in OA.^109^ Recent studies in OA animal models such as DMM and ACLT have shown that nanodrug systems outperform free‐form drugs, particularly in subchondral bone targeting, synovial tissue penetration, and dual‐targeting interventions.^110^, ^111^, ^112^ In vivo data demonstrated that nanocarrier‐mediated drugs exhibited prolonged retention within the OA‐affected knee joint and higher accumulation in subchondral bone. These effects strongly correlated with local suppression of CatK expression, maintenance of cartilage thickness, and downregulation of inflammatory cytokines, highlighting the biological rationale for nanocarrier‐based targeted delivery (Table 2).^113^

**TABLE 2:** Comparative chart of structures and functions of various types of nanocarriers.

Name	Liposomes	PLGA nanoparticles	Fe₃O₄ nanoparticles	Self‐assembling peptides	Hydrogels	Exosome membranes
Structure	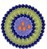	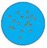	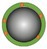	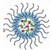	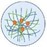	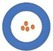
Size	20–80 nm to 1–5 μm	100–300 nm	10–50 nm	10–100 nm	100–500 nm	~100 nm
Release mechanism	Passive diffusion, membrane fusion, or triggered release (pH/enzyme‐sensitive)	Hydrolysis‐driven degradation	Passive diffusion or magnetothermal‐triggered release	Enzymatic cleavage or pH‐responsive structural changes	Swelling‐controlled diffusion or chemical bond cleavage (weeks‐long sustained release)	Membrane fusion or endocytosis‐mediated delivery (inherent homing ability)
Biocompatibility	High	High	Moderate	High	High	High
Delivery route	Intra‐articular injection	Intra‐articular injection	Implantation	Intra‐articular injection	Intra‐articular injection	Intra‐articular injection
Targeting strategies	Enhanced Permeability and Retention (EPR) effect in tumors/inflammatory tissues Surface modification with antibodies (e.g., anti‐HER2), ligands (e.g., folate), or peptides (e.g., RGD), Magnetic liposomes guided by external fields	EPR effect; PEGylation prolongs circulation, Conjugation with transferrin or pH‐responsive coatings for tumor targeting	Accumulation at target sites via external magnets Combined with liposomes/PLGA for dual targeting and controlled release	Incorporation of tumor‐homing motifs (e.g., RGD for integrin binding) Release triggered by tumor‐specific enzymes (e.g., MMPs)	Direct implantation into diseased tissues (e.g., tumors or joints) Embedding targeted nanoparticles (e.g., liposomes) for dual‐stage release	Tissue‐specific targeting via surface proteins (e.g., CD47 for immune evasion) Fusion with targeting ligands (e.g., folate) or antibodies
Advantages	High encapsulation efficiency for hydrophilic/hydrophobic drugs; flexible functionalization	Sustained release; FDA‐approved for drug delivery	Theranostic capabilities (MRI imaging /photo‐thermal therapy)	Precise molecular design; modular functionality	Mechanical support; customizable degradation rates	Natural biocompatibility; evasion of immune clearance
Limitations	Poor stability (oxidation/leakage); rapid clearance by macrophages without PEGylation	Low drug‐loading capacity; weak intrinsic targeting	Potential long‐term toxicity from iron accumulation	Challenges in large‐scale production; limited drug‐loading capacity	Low drug‐loading efficiency; limited systemic delivery potential	Low drug‐loading efficiency; complex isolation/purification processes
Reference	PMID: 37258510, PMID: 37864947	PMID: 37794470	PMID: 38877353	PMID: 40963906, PMID: 22818981	PMID: 37802985, PMID: 37258510	PMID:35836795, PMID: 33243424

Currently applied nanocarriers in OA treatment include: Lipid‐based nanoparticles (e.g., liposomes, solid lipid nanoparticles)^114^, ^115^; Polymeric nanoparticles (e.g., PLGA, PEG‐PLGA, PCL and related polymers)^36^; Inorganic nanoplatforms (e.g., Fe_3_O_4_, MnO_2_, SiO_2_)^109^; Self‐assembling nanostructures (e.g., peptide‐based systems, hydrogels, DNA nanostructures)^114^, ^116^, ^117^, ^118^; Biomimetic materials (e.g., exosomes, cell membrane‐coated systems).^119^, ^120^ The design of these carriers should be tailored to the OA microenvironment and meet criteria such as excellent biocompatibility, injectability, controlled degradability and release, target recognition capacity, and stimuli‐responsiveness. Particle sizes are generally controlled within the 100–200 nm range to balance joint permeability with intra‐articular retention.^121^

### 4.2 Targeted functionalization strategies: achieving cell/tissue‐specific recognition

Based on established nanocarrier architectures, targeted functionalization plays a pivotal role in achieving precise drug delivery. Researchers have modified nanocarrier surfaces with specific ligands to enable selective localization to osteoclasts or chondrocytes. For instance, D‐Asp_8_ peptides bind to calcium‐rich bone sites associated with osteoclasts, while CD44 ligands such as hyaluronic acid or RGD peptides target integrin receptors on the chondrocyte surface.^122^, ^123^ These strategies have been validated in both primary osteoclast and OA chondrocyte models, demonstrating significantly enhanced cellular uptake and increased local drug concentrations.^121^

Furthermore, biomimetic modifications—such as coating nanoparticles with exosomal or macrophage‐derived membranes—offer intrinsic cellular fusion capabilities and immune evasion properties, thereby enhancing lesion‐specific targeting.^124^, ^125^ Dual or multivalent ligand designs (e.g., RGD + D‐Asp_8_ co‐functionalization) have enabled simultaneous targeting of bone and cartilage, providing a structural basis for integrated OA interventions (Figure S2).^121^

### 4.3 Microenvironment‐responsive release mechanisms: enhancing site‐specific drug delivery

The OA lesion microenvironment is characterized by distinct physicochemical and biochemical features compared with healthy joints (pH ≈7.4). These include an acidic milieu (pH 6.0–6.8), elevated expression of proteolytic enzymes such as Cathepsin K (CatK) and MMP‐13, and accumulation of ROS.^126^, ^127^ The acidic environment primarily arises from anaerobic glycolysis in inflammatory and chondrocyte populations, as well as proton release during osteoclastic bone resorption. Stimuli‐responsive nanoplatforms have been designed to exploit these pathological microenvironmental cues by incorporating pH‐sensitive linkages (e.g., hydrazone or cis‐aconityl bonds), enzyme‐cleavable sites (e.g., CatK‐specific peptide sequences), or ROS‐labile moieties (e.g., diselenide or thioether bonds). Such designs enable site‐specific drug release within the OA lesion. For instance, PLGA–hydrazone nanoparticles, with a cleavage threshold of approximately pH 6.5, were shown in DMM mouse models to achieve precise CatK inhibitor release, thereby effectively suppressing bone resorption and maintaining cartilage thickness.^128^

Multimodal systems that integrate pH and ROS sensitivity or combine multiple enzymatic triggers allow for both spatial and temporal control over drug release. Dual‐enzyme‐responsive platforms targeting CatK and MMP‐13 can sequentially release two agents, enhancing synergistic therapeutic outcomes. Organoid‐based studies further confirmed that such systems could accurately detect pathological cues within 3D microenvironments, simulate dynamic synovial conditions, and achieve sustained and controllable drug release (Figure S3).^129^

### 4.4 Multi‐target co‐delivery and interface‐regulating systems

As OA is a disorder of bone‐cartilage coupling imbalance, single‐target delivery approaches often fail to achieve comprehensive therapeutic effects. In response, multi‐target co‐delivery systems have been developed, commonly employing: (1) dual‐ligand surface modification strategies (e.g., RGD + D‐Asp_8_); (2) core‐shell architectures (e.g., CatK inhibitor in the core, MMP‐13 inhibitor in the shell); and (3) layer‐by‐layer assembled nanoplatforms for sequential, stimuli‐responsive drug release. Such systems have demonstrated multiple therapeutic advantages in both DMM models and OA organoid platforms, including simultaneous improvement in osteoclast and chondrocyte markers, enhanced stability of the osteochondral interface, and reduced inflammatory scores.^129^

Additionally, certain materials—such as calcium phosphate and strontium peptide dual‐network hydrogels—exhibited both drug delivery and interface regenerative functions. These platforms promoted structural reconstruction and redifferentiation at the bone‐cartilage junction, expanding the potential of nanotechnology for integrated repair‐and‐regulation strategies in OA.^121^

### 4.5 Smart feedback systems and nucleic acid drug platforms

The frontier of nanodrug delivery systems for OA is advancing toward intelligent and personalized therapeutic designs. One key direction is the development of intelligent feedback‐closed‐loop systems, which integrate both diagnostic and therapeutic functionalities. For example, nanoparticles incorporating pH‐ or ROS‐responsive MRI contrast agents or near‐infrared (NIR) fluorescent probes can not only visualize drug distribution within OA lesions but also autonomously trigger drug release upon sensing specific pathological cues, such as an inflammatory threshold. This enables the creation of a “sense–release–feedback” autonomous therapeutic loop.^130^

At the same time, the combination of nucleic acid therapeutics (such as siRNA and miRNA) with nanoplatforms provides a powerful approach for precise inhibition of pathogenic gene expression. For instance, co‐delivery of a CatK inhibitor and HIF‐2α siRNA has demonstrated synergistic efficacy in animal OA models, simultaneously suppressing bone resorption and cartilage degradation.^131^, ^132^ The integration of intelligent feedback systems, nucleic acid delivery, and artificial intelligence collectively forms the foundation of next‐generation precision nanomedicine for OA.

In summary, the integration of smart feedback systems and nucleic acid‐based therapies offers a promising direction for multi‐level modulation of communication axes in OA, paving the way for highly integrated platforms that span from signal sensing to controlled drug release (Figure S4).

## 5 CLINICAL TRANSLATION BARRIERS AND FUTURE DIRECTIONS

### 5.1 Heterogeneity of OA and limitations in translating drug delivery systems

OA is not a single disease entity but a highly heterogeneous degenerative condition, with its clinical phenotype varying by lesion site (e.g., knee, hip, hand), age, gender, body mass index, genetic background, and accompanying metabolic conditions such as diabetes and hyperlipidemia. This heterogeneity directly influences cellular involvement, inflammatory intensity, and the status of the joint microenvironment during disease progression.^133^ While nanodelivery systems have demonstrated promising efficacy in standardized murine models, their translational performance in human OA patients remains uncertain. In complex human joint environments, delivery efficiency may decline, targeting accuracy may weaken, and stimuli‐responsive mechanisms may fail. For example, in OA patients with severe synovial fibrosis, increased synovial fluid viscosity significantly impairs nanoparticle diffusion, reducing drug penetration and accumulation.^134^

Common OA models such as DMM and ACLT often utilize young, healthy mice or rabbits that lack critical features of human OA, including metabolic dysfunction and immunosenescence. As a result, the targeting precision, tissue penetration capacity, and release kinetics of drug delivery systems may differ substantially between animal models and human patients. For instance, nanocarriers exhibiting robust pH responsiveness in mice may fail in the human joint cavity due to insufficient pH gradients.^135^

Moreover, the thickness of mouse cartilage is approximately 1/70 that of human cartilage,^136^ making its drug distribution patterns poorly representative of clinical conditions. Therefore, it is essential to validate nanodelivery platforms across more physiologically relevant models to ensure translational viability. Supporting this, recent studies showed that pH‐responsive nanoparticles released drugs efficiently in DMM models but displayed significantly slower release rates in large‐animal models and human cartilage explants, highlighting the critical role of interspecies physiological differences and underscoring the importance of cross‐species validation.^137^

### 5.2 Organoid and large‐animal models as bridging platforms

OA organoids are three‐dimensional tissue‐engineered models constructed from human‐derived cells, typically composed of co‐cultured synovial, cartilage, and subchondral bone cells. These systems simulate key features of OA pathology, including intercellular communication, mechanical stimulation, and the inflammatory microenvironment.^138^, ^139^ Current organoid platforms are often built using natural hydrogels such as gelatin methacrylate (GelMA), decellularized extracellular matrix, or synthetic polymers like PEGDA. These scaffolds are embedded with primary cells derived from human OA synovium, cartilage, and bone marrow to reconstruct the tissue interface and microstructural characteristics.^140^ Some platforms have incorporated microfluidic chip systems to replicate synovial shear stress and nutrient gradients, thereby enhancing dynamic response capabilities.^141^

Studies have demonstrated that GelMA‐based hydrogels exhibit excellent biocompatibility and tunable mechanical properties, making them well‐suited for evaluating drug release kinetics and spatial distribution within organoids.^138^, ^142^ These platforms offer several unique advantages for assessing nanodelivery performance: (1) they recapitulate human pathological features, enhancing translational relevance; (2) they enable high‐throughput efficacy evaluation via fluorescence imaging and single‐cell sequencing; and (3) they allow for controlled experimental variables to delineate drug mechanisms of action.^143^ Recent findings indicate that pH/ROS dual‐responsive nanoplatforms tested in OA organoids demonstrated drug release profiles and cellular uptake patterns more closely aligned with human clinical behavior.^144^ In organoid drug delivery studies, these systems were shown to reflect synovial viscosity changes and intra‐articular drug retention times, while reproducing inflammation‐associated cytokine release and cartilage matrix responses consistent with human tissue, thereby confirming their reliability and translational potential in nanotherapeutic evaluation.

After constructing correlation hypotheses at the organoid level, small‐animal models serve as indispensable tools for elucidating pathological mechanisms and validating causal relationships, owing to their complete in vivo physiological systems. The mouse DMM (destabilization of the medial meniscus) model is the most commonly used. By surgically disrupting the meniscotibial ligament, it reproducibly induces classic OA phenotypes—such as superficial cartilage erosion, subchondral bone sclerosis, and osteophyte formation—within 8–12 weeks. The pathological progression observed in this model closely mirrors the “cartilage–bone interplay” mechanism of human primary OA.^145^ The rat ACLT (anterior cruciate ligament transection) model, with its larger joint size, is more suitable for intra‐articular injection and drug‐delivery studies. It also exhibits synovial inflammation and synovial fluid compositional changes that accurately recapitulate the pathophysiological features of post‐traumatic OA.^60^ However, these models have inherent limitations. Rodent–human differences in age, sex, and gait affect disease dynamics; rats do not develop spontaneous OA, limiting their utility for studying idiopathic OA progression. Furthermore, the small size of rodent joints results in limited available tissue, constraining histological and molecular analyses.^146^

In contrast, large‐animal models offer significantly higher fidelity in simulating human OA phenotypes. Their major advantages lie in anatomical similarity, pathological consistency, and the ability to model comorbid conditions. From an anatomical and biomechanical perspective, the canine knee joint closely resembles that of humans in terms of flexion–extension range and load distribution.^147^ In an ACLT dog model, pharmacokinetic studies of nanocarriers revealed joint retention times, release kinetics, and inflammatory responses that closely mirrored human data.^148^ Additionally, the model demonstrated strong concordance with human OA across multiple outcome measures, including joint mobility restoration, cytokine expression profiles, synovial thickening, and cartilage fissure scores—thereby increasing its scientific value as a late‐stage validation platform.^147^ While large‐animal studies entail higher costs and longer experimental durations, they offer irreplaceable scientific value in advancing clinical translation. These models are particularly well‐suited for evaluating biodistribution, immune tolerance, and safety of nanodelivery systems.^149^

### 5.3 Platform extension potential: From OA to inflammation, regeneration, and oncology

OA nanodelivery systems possess modular and highly reconfigurable structural features, providing a material foundation for their extension to other chronic disease applications.^150^ Previous studies have adapted these platforms for diseases such as rheumatoid arthritis (RA, targeting the JAK pathway), ankylosing spondylitis (AS, via RNAi targeting IL‐17/IL‐23 pathways), and fracture repair (incorporating osteogenic factor‐loaded nanosystems). By modifying targets, functional ligands, and stimulus‐responsive mechanisms, these platforms demonstrate excellent cross‐indication adaptability.^151^

Moreover, such platforms have been expanded into areas including tumor site‐specific drug release, brain injury repair, and cartilage tissue engineering. For instance, in tumor models, systems combining pH‐ and ROS‐responsiveness enabled controlled drug release under oxidative tumor microenvironments.^152^ In the context of bone regeneration, composite nanosystems carrying growth factors showed promise in reconstructing the bone–cartilage interface and promoting both osteogenesis and chondrogenesis.^153^ Collectively, OA nanoplatforms are evolving into versatile intervention systems for inflammation–destruction–repair pathways, with a robust technological foundation for structural migration across tissues and pathological mechanisms.

## 6 CONCLUSION AND OUTLOOK

### 6.1 Paradigm shift from mechanistic understanding to therapeutic intervention

This review systematically elucidates the theoretical basis of the osteoclast–chondrocyte communication axis as a core driving mechanism of OA and summarizes small‐molecule inhibitors targeting key nodes along this axis, such as RANKL, CatK, and HIF‐2α. However, the delivery limitations of conventional small‐molecule drugs and the constraints of single‐target therapies have driven a paradigm shift toward “nanocarrier‐assisted multi‐target synergistic intervention.” Functional and intelligent nanodrug delivery systems—by enhancing targeting specificity, enabling stimuli‐responsive release, and supporting combination delivery—have become the critical bridge that translates fundamental mechanistic insights into effective therapeutic strategies.

### 6.2 Scientific questions and technical bottlenecks for the future

Despite advances in the study of the OA communication axis, several critical scientific questions remain unresolved. How can we dynamically characterize the activation patterns of the communication axis at the patient level? Current tools such as scRNA‐seq, multi‐omics integration, and long‐term clinical follow‐up remain insufficient. (1) Do different OA subtypes exhibit distinct activation patterns of the communication axis? Should subtype‐specific target screening be prioritized? (2) Are there yet undiscovered regulatory molecules or non‐coding RNA axes involved? and (3) On the technical front, existing nanodelivery systems still face challenges related to material controllability, biological stability, and scalable manufacturing. Given the anatomical complexity of OA, drug delivery must accommodate multiple tissues—synovium, cartilage, and subchondral bone—yet current evaluation systems lack standardization. Future efforts should focus on developing an integrated “function‐assessment‐regulation” framework encompassing therapeutic efficacy, biocompatibility, and safety indicators to facilitate regulatory approval, clinical translation, and scalable implementation.

### 6.3 From mechanism to clinic: Constructing a translational bridge

To achieve a closed‐loop pipeline from molecular mechanism to clinical application, a five‐tier translational pathway must be established, centered on “mechanism validation—model screening—platform delivery—clinical assessment”: (1) Cellular and molecular stage: Develop co‐culture systems integrating osteoclasts, chondrocytes, and synoviocytes, and use multi‐omics approaches to identify communication axis targets. (2) Small animal validation: Utilize DMM and ACLT models to assess structural degradation, inflammatory responses, and therapeutic windows. (3) OA organoid models: Reconstruct 3D synovium‐cartilage‐bone interfaces to evaluate spatial drug distribution and target specificity. (4) Large animal verification: In canine or ovine models, monitor drug retention, cartilage penetration, and functional recovery. (5) Phase I clinical trial: Focus on safety profiling, dose escalation, and target occupancy as primary endpoints to initiate clinical translation.

## AUTHOR CONTRIBUTIONS

Enbo Zhang and Bo Li conceived and designed the study. Bowen Gu, Enbo Zhang, and Bo Li performed the experiments. Chi Ma and Xiaohe Fan analyzed the data. Enbo Zhang and Bo Li wrote the manuscript. All authors reviewed and approved the final version of the manuscript.

## FUNDING INFORMATION

None.

## CONFLICT OF INTEREST STATEMENT

The authors declare no conflict of interest.

## Supporting information


**FIGURE S1.** Challenges in small‐molecule drug delivery and strategies for combinatorial intervention.
**FIGURE S2.** Schematic representation of targeted nanodelivery strategies for OA therapy.
**FIGURE S3.** Microenvironment‐responsive release and multi‐enzyme synergy mechanisms.
**FIGURE S4.** Closed‐loop schematic of an intelligent feedback‐based nanodelivery platform.
**TABLE S1.** Representative experimental data demonstrate the morphology of the nanocarriers, the osteoarthritis (OA) pathology, and the therapeutic effects.

## Data Availability

All data generated or analyzed during this study are included in this article and/or its supplementary material files. Further enquiries can be directed to the corresponding author.
