# Supplementary material for: Bridging the gap in OA therapeutics: Bioengineered strategies to target osteoclast–chondrocyte crosstalk
Source: Bioeng Transl Med. 2026 Jan 11;11(3):e70107. doi: 10.1002/btm2.70107 (PMC13247419; doi:10.1002/btm2.70107)
Supplement: Supplementary file 1 — FIGURE S1. Challenges in small‐molecule drug delivery and strategies for combinatorial intervention. FIGURE S2. Schematic representation of targeted nanodelivery strategies for OA therapy. FIGURE S3. Microenvironment‐responsive release and multi‐enzyme synergy mechanisms. FIGURE S4. Closed‐loop schematic of an intelligent feedback‐based nanodelivery platform. TABLE S1. Representative experimental data demonstrate the morphology of the nanocarriers, the osteoarthritis (OA) pathology, and the therapeutic effects. [file BTM2-11-e70107-s001.docx]

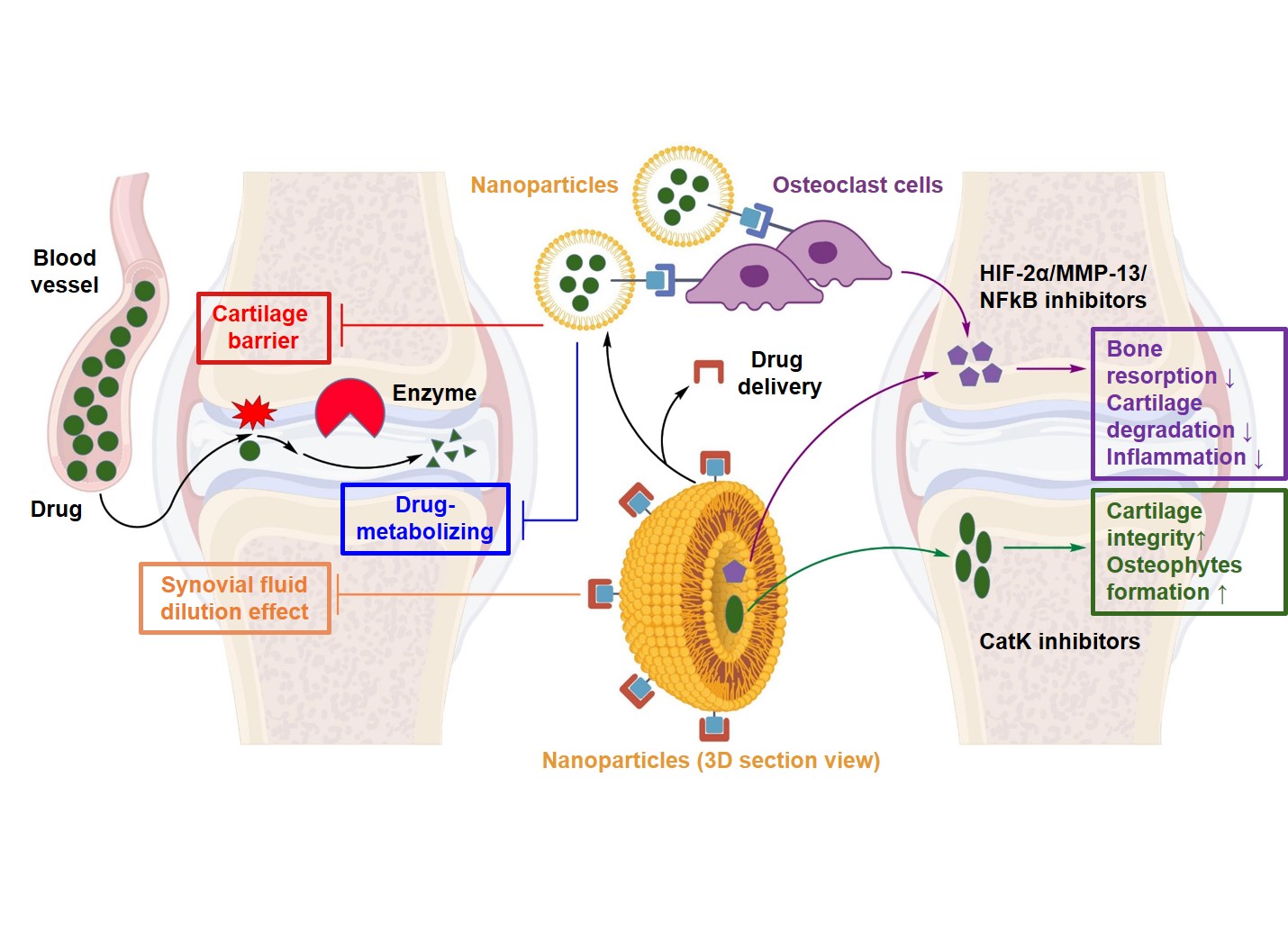


**Figure S1. Challenges in small-molecule drug delivery and strategies for combinatorial intervention.**


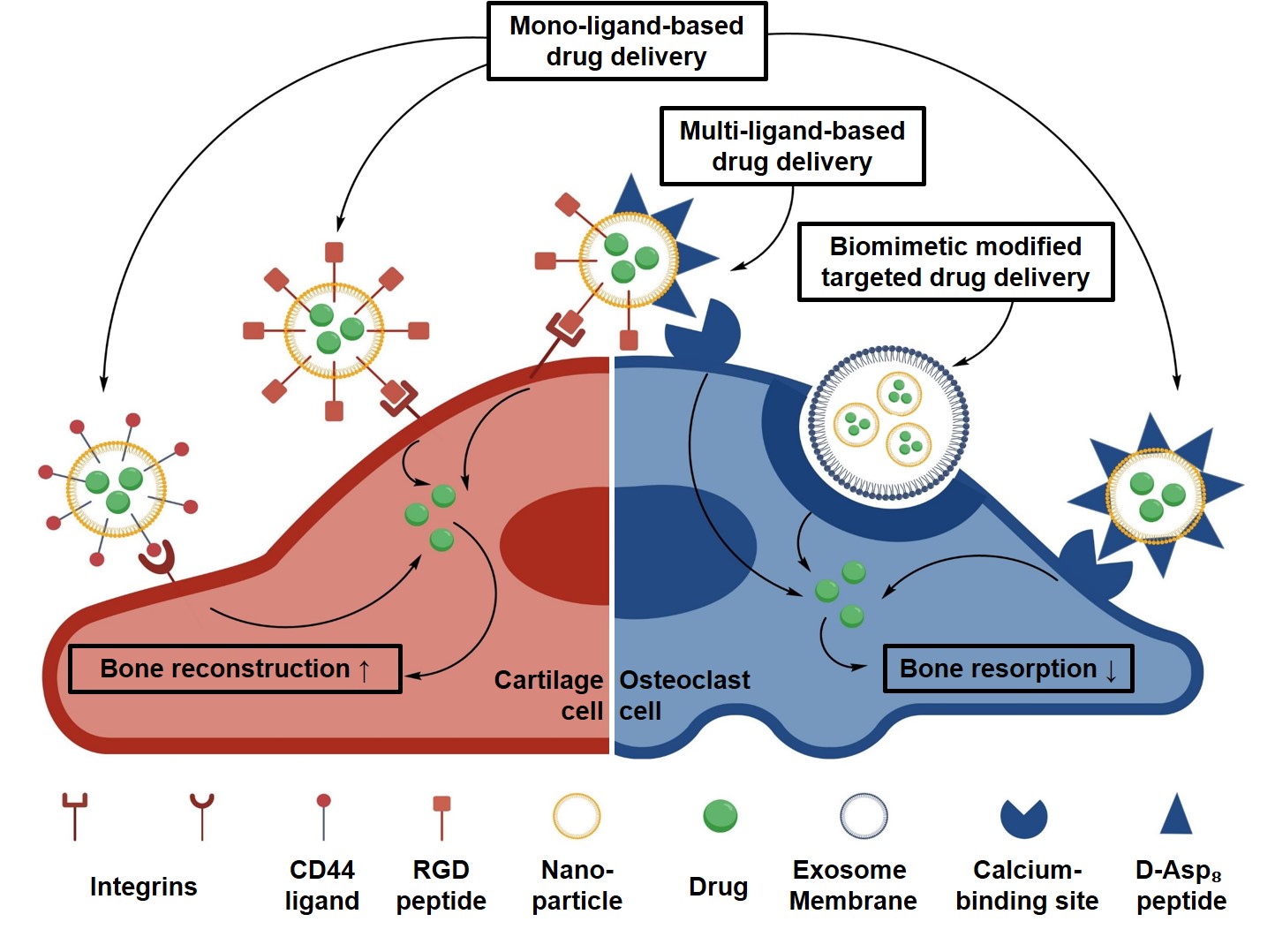


**Figure S2. Schematic representation of targeted nanodelivery strategies for OA therapy.**


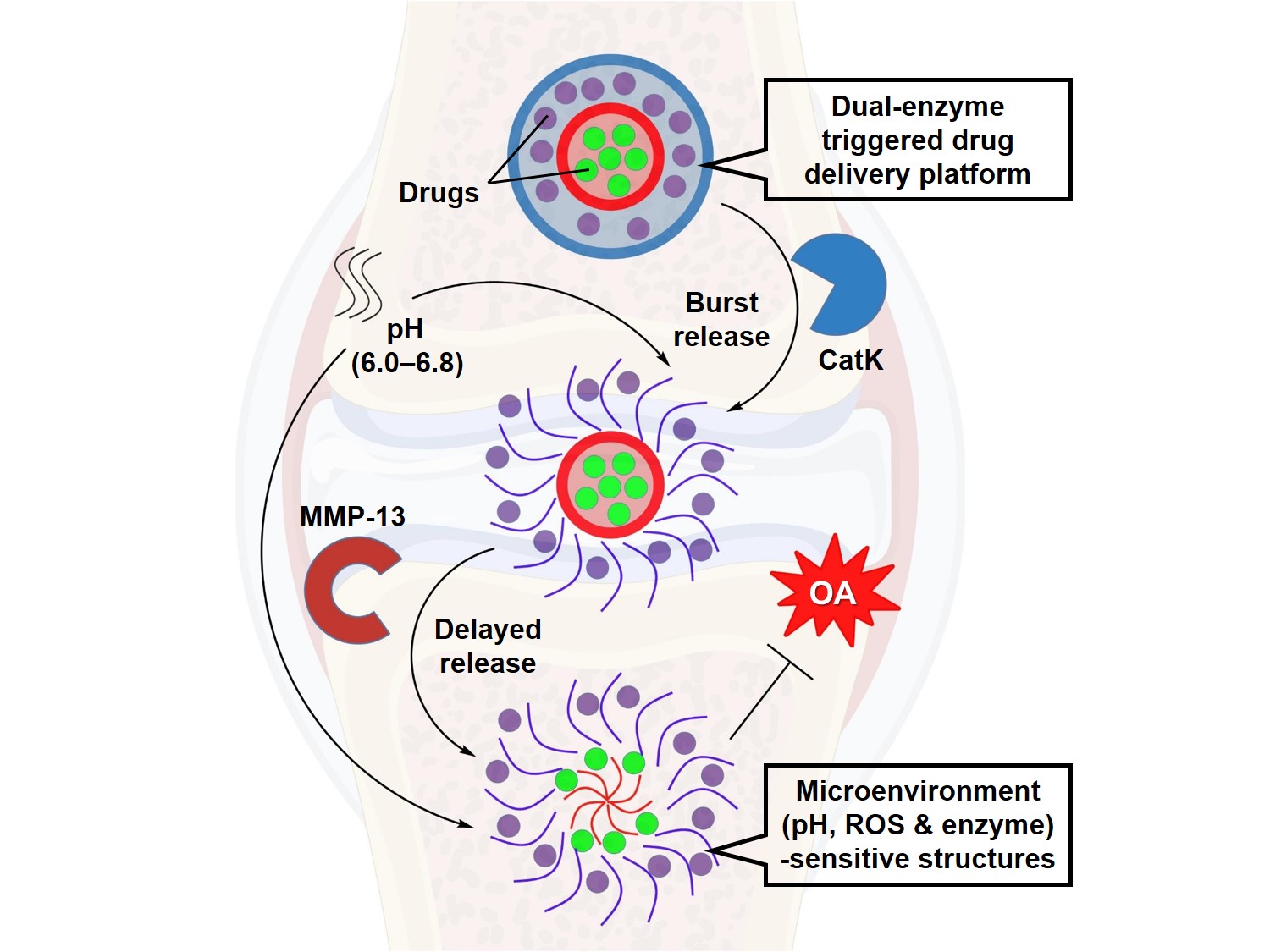


**Figure S3. Microenvironment-responsive release and multi-enzyme synergy mechanisms.**


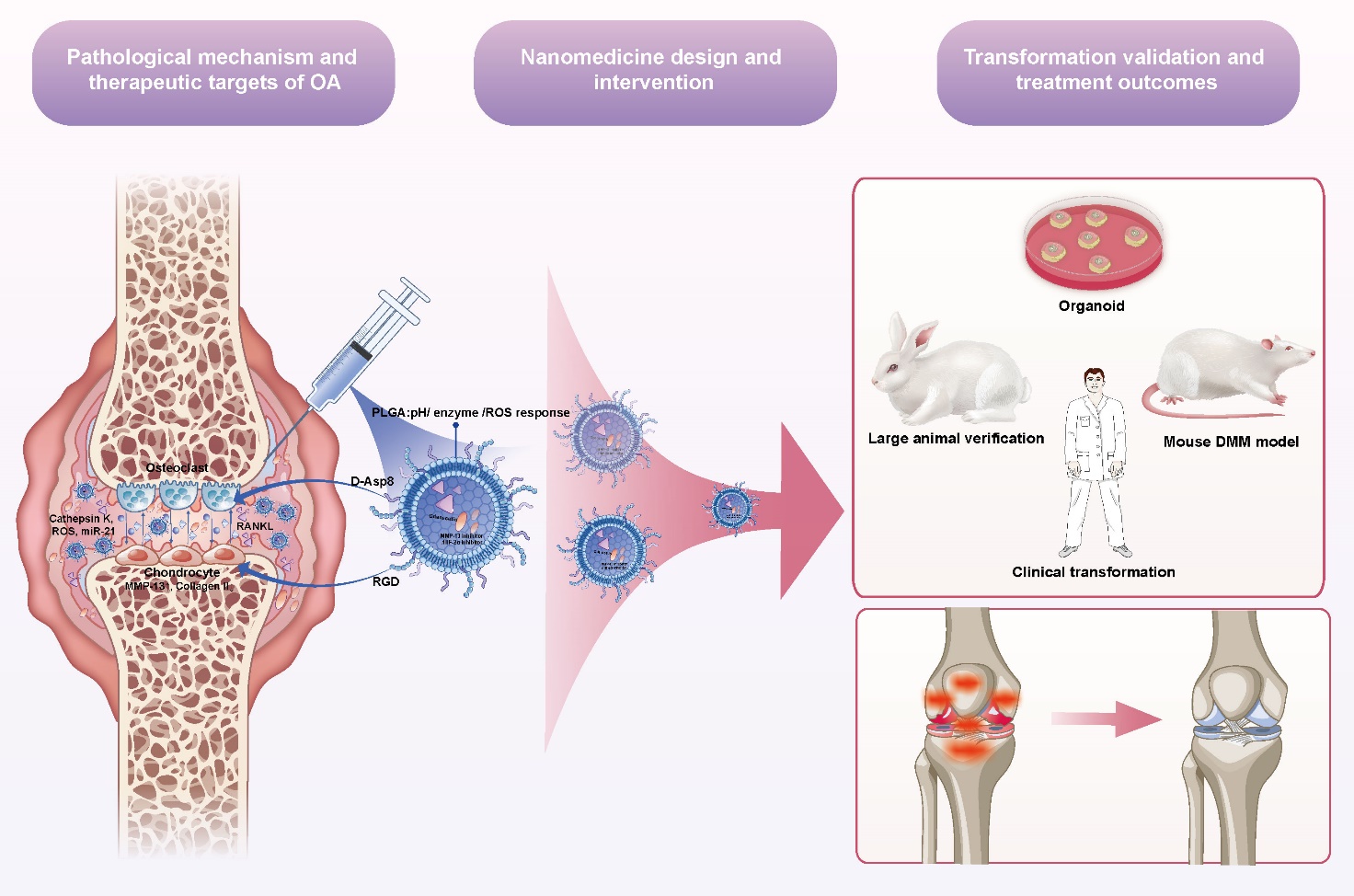


**Figure S4. Closed-loop schematic of an intelligent feedback-based nanodelivery platform.**

**Table S1. Representative experimental data demonstrate the morphology of the nanocarriers, the osteoarthritis (OA) pathology, and the therapeutic effects.**

| **Name** | **Liposomes‌** | **PLGA**  **Nanoparticles** | **Fe₃O₄**  **Nanoparticles** | **Self-Assembling Peptides** | **Hydrogels‌** | **Exosome**  **Membranes** |
| --- | --- | --- | --- | --- | --- | --- |
| **TEM images of nanocarriers** | **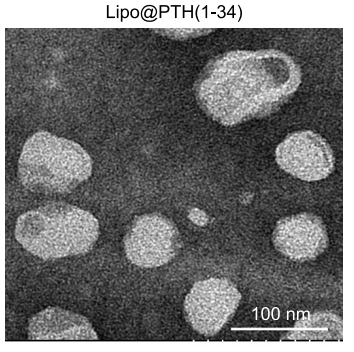** | **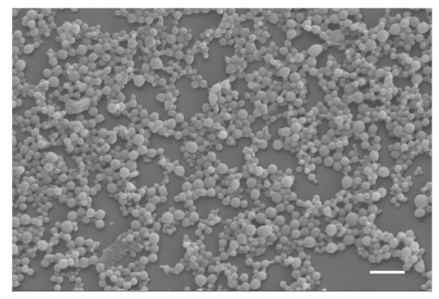** | **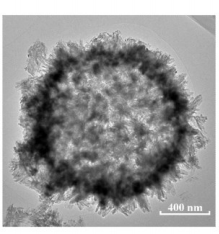** | **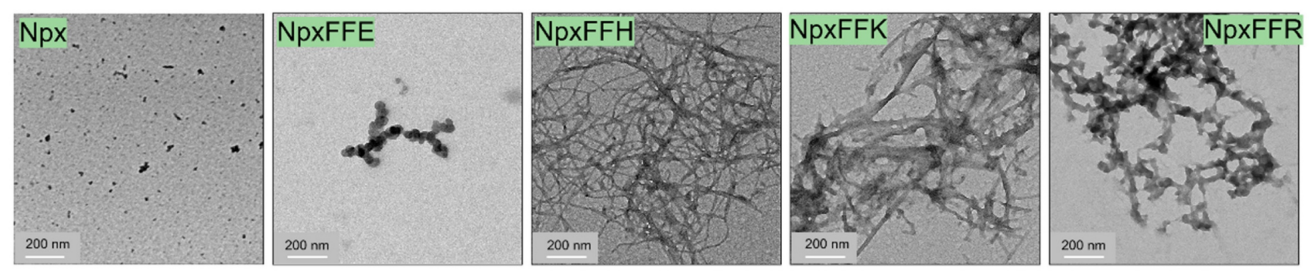** | **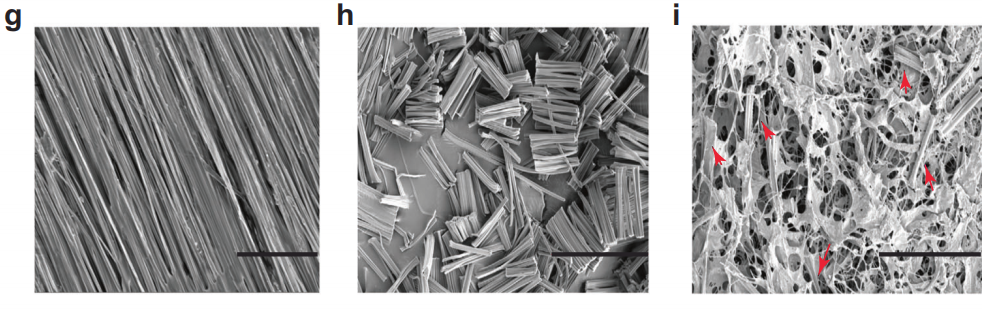** | **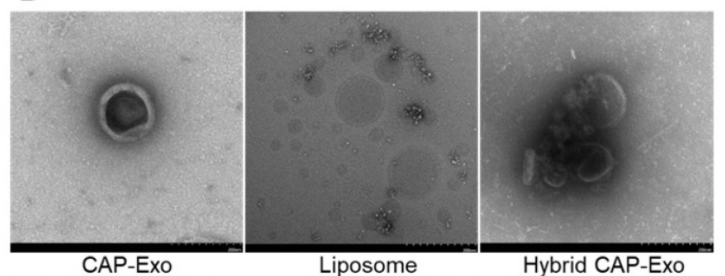** |
| **In vivo fluorescence imaging of nanocarrier biodistribution** | **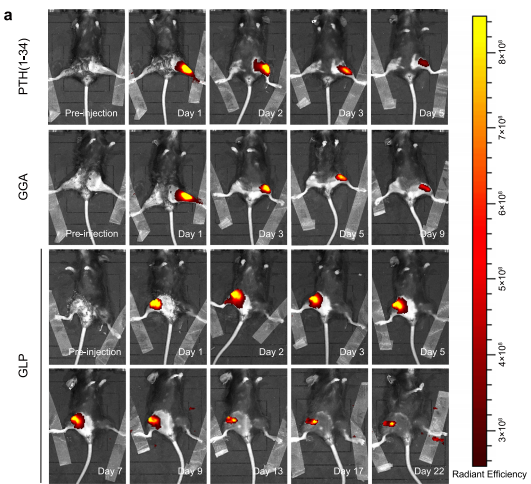** | **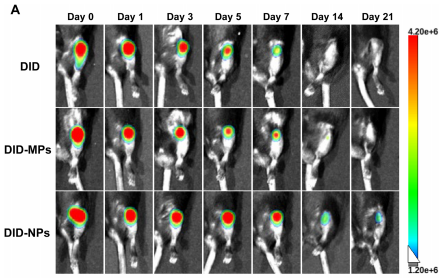** | **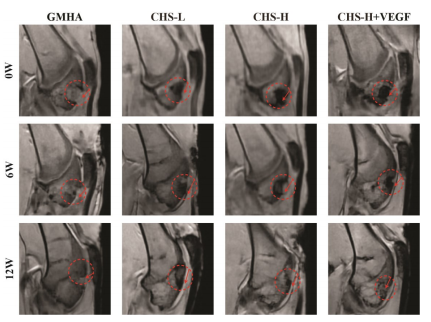** | **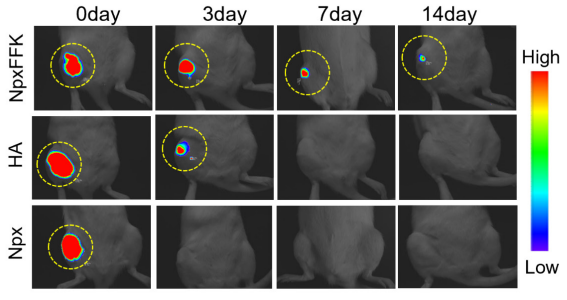** | **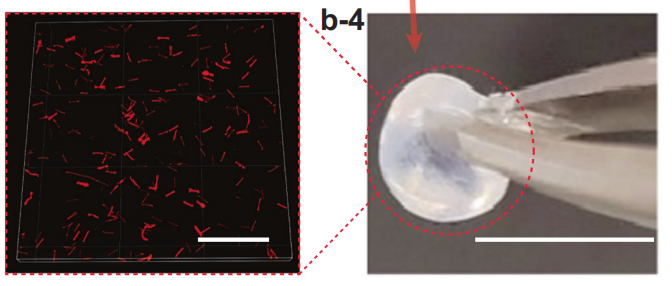** | **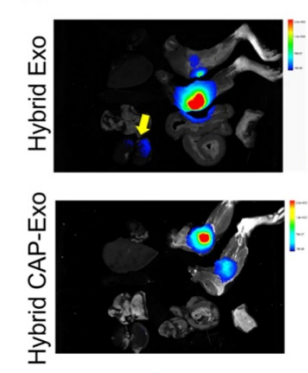** |
| **Histological images of cartilage repair post-treatment** | **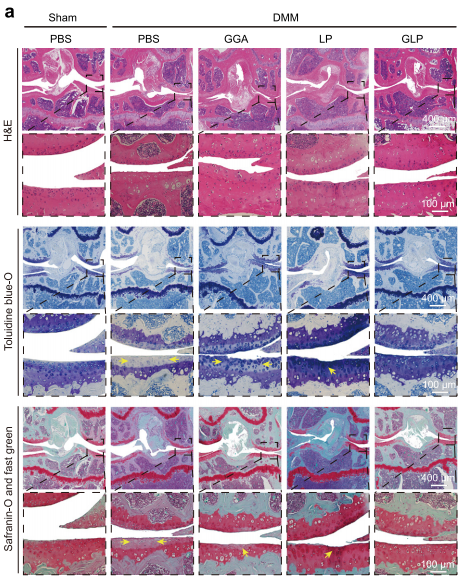** | **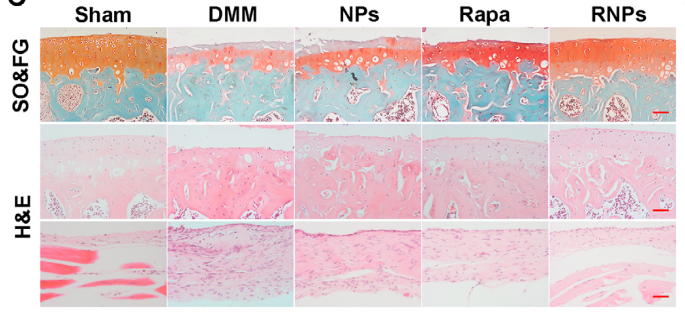** | **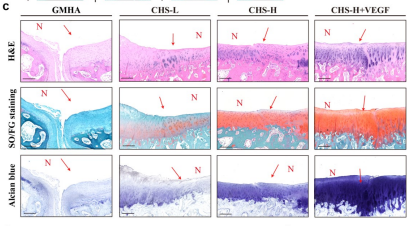** | **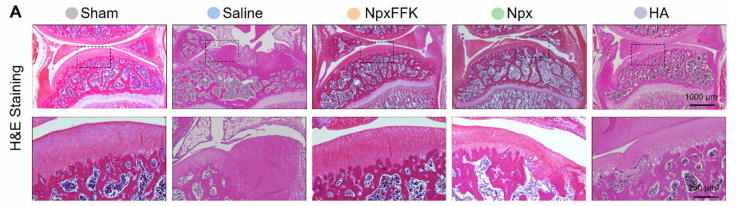** | **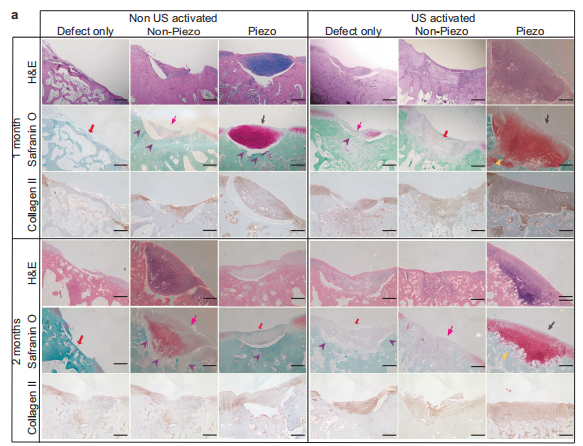** | **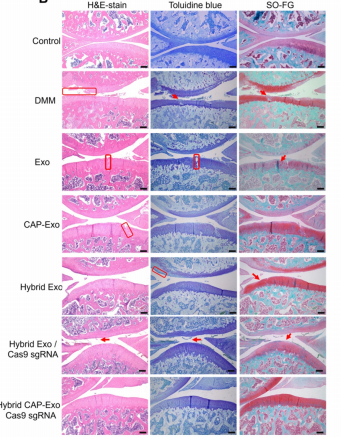** |
| Reference | PMID: 37258510 | PMID: 37794470 | PMID: 38877353 | PMID: 40963906 | PMID: 37802985 | PMID:35836795 |
